# Coordination Ring-Opening Polymerization of Cyclic Esters: A Critical Overview of DFT Modeling and Visualization of the Reaction Mechanisms

**DOI:** 10.3390/molecules24224117

**Published:** 2019-11-14

**Authors:** Ilya Nifant’ev, Pavel Ivchenko

**Affiliations:** 1 Chemistry Department, M.V. Lomonosov Moscow State University, 1 Leninskie Gory Str., Building 3, 119991 Moscow, Russia; 2 A.V. Topchiev Institute of Petrochemical Synthesis RAS, 29 Leninsky Pr., 119991 Moscow, Russia

**Keywords:** density functional theory, lactones, lactides, cyclic carbonates, cyclic phosphates, ring-opening polymerization, coordination catalysis

## Abstract

Ring-opening polymerization (ROP) of cyclic esters (lactones, lactides, cyclic carbonates and phosphates) is an effective tool to synthesize biocompatible and biodegradable polymers. Metal complexes effectively catalyze ROP, a remarkable diversity of the ROP mechanisms prompted the use of density functional theory (DFT) methods for simulation and visualization of the ROP pathways. Optimization of the molecular structures of the key reaction intermediates and transition states has allowed to explain the values of catalytic activities and stereocontrol events. DFT computation data sets might be viewed as a sound basis for the design of novel ROP catalysts and cyclic substrates, for the creation of new types of homo- and copolymers with promising properties. In this review, we summarized the results of DFT modeling of coordination ROP of cyclic esters. The importance to understand the difference between initiation and propagation stages, to consider the possibility of polymer–catalyst coordination, to figure out the key transition states, and other aspects of DFT simulation and visualization of ROP have been also discussed in our review.

## 1. Introduction

**Scheme 1:**
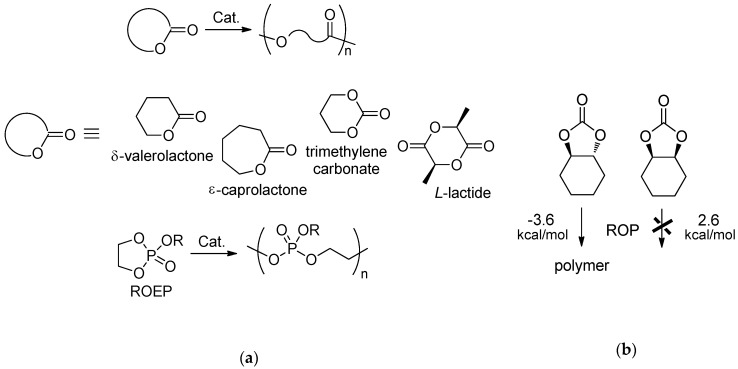
(**a**) Ring-opening polymerization of cyclic esters (formula of typical representatives are given) and alkyl ethylene phosphates (ROEP); (**b**) Bicyclic carbonate isomers that are active and inactive in ROP owing to thermodynamic criteria [31].

**Figure 1:**
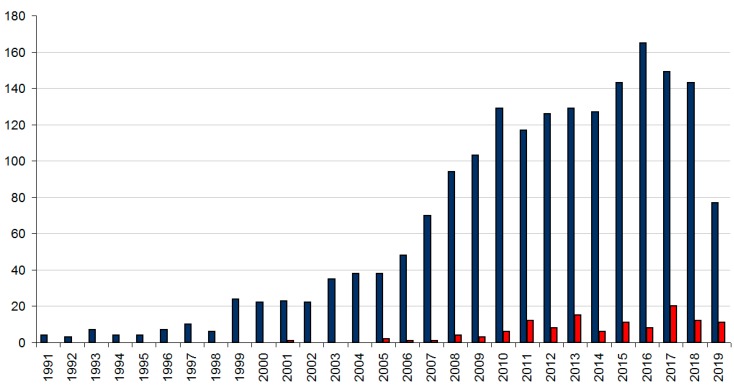
Annual numbers of publications on the topic of ring-opening polymerization of cyclic esters (blue) and numbers of publications that include DFT modeling of ROP (red), Web of Science data (Thomson-Reuters, October 30, 2019).

Biodegradable and biocompatible polymers have attracted the most attention of the researchers in the last two decades [1,2,3,4,5]. This interest has been driven by current social and economic motivations and challenges. The replacement of traditional petroleum-based plastics [4,6] and diversity in biomedical applications [1,4] are the main areas of the use of biodegradable polymers. Linear aliphatic polyesters, such as polylactones, polylactides, polycarbonates, poly-(1,4-dioxan-2-one), and poly(ethylene phosphate)(s) can be synthesized via catalytic ring-opening polymerization (ROP) of the corresponding cyclic monomers (Scheme 1a) [6,7,8,9,10,11,12,13]. ROP of cyclic esters became a genuine hot topic in the end of 1990 s (Figure 1). Catalysis plays a primary role in the synthesis of polyesters [14]. To date, various ROP mechanisms and catalysts have been studied and comprehensively reviewed [15,16,17,18,19,20,21,22,23,24,25,26,27,28,29,30]. The density functional theory (DFT) methods have been applied for the modeling of the ROP successfully and fruitfully. However, there was no attempt to integrate and review the fragmented data on DFT modeling of ROP of various cyclic substrates in the frameworks of qualitatively different reaction mechanisms. The present review is a result of our effort to fill the gap.

In line with different types of cyclic monomers, the material presented in the review is divided into five main parts. The first three parts are concerned with DFT modeling of homopolymerization of conventional monomers such as lactones, cyclic carbonates, and lactides. In addition to discussion of the ROP of traditional cyclic esters, in part four we address the issue of the polymerization of cyclic ethylene phosphate monomers [12,13] (ROEP in Scheme 1) due to similarity of the reaction mechanisms for cyclic lactones, lactides, carbonates and phosphates, related compounds (including ethylene phosphates). The random copolymerization of cyclic substrates that differ by their chemical nature have been discussed in the final part.

Before beginning discussions regarding the simulations of the ROP mechanisms in terms of the reaction *kinetics* (the nature of the reaction complexes, intermediates and transition states), the key aspect of the ROP *thermodynamics* should be briefly discussed. Obviously, the investigation of the reactions presented in Scheme 1 should include the pre-synthetic analysis: the choice of the cyclic monomer which is suitable for controlled catalytic ROP should take into account the total change of the free energy during the ring-opening [32]. The estimation of the value of the free energy is a most simple application of DFT modeling, and the negative change of the free energy (exergonic process) is a helpful criterion for the selection of “good” monomers. Such DFT estimation was successfully applied, for example, in the study of the novel substrates, bicyclic carbonates [31] (Scheme 1b).

**Scheme 2:**
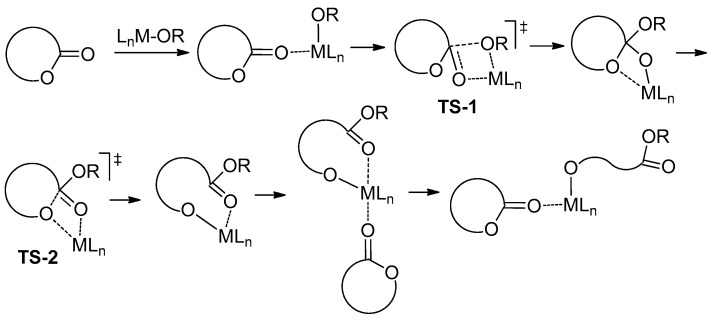
The draft of coordination-insertion mechanism of ROP of cyclic esters, metal alkoxide as an initiator.

While on the surface the mechanisms of ROP catalyzed by metal complexes seem to be diverse and cumbersome, most of them could be described in the framework of the common sequence of stationary points (SP) and transition states (TS) named *coordination-insertion* mechanism (Scheme 2) [11,22,23,24]. The metal atom plays the role of the electrophilic catalytic center that coordinate the molecule of the cyclic ester. This coordination results in activation of the carbon atom of the carbonyl group for cyclic esters or phosphorus atom for cyclic phosphates. The initiator (alkoxy, amino, alkyl etc.) can be directly bonded or weakly coordinated with the metal. Such dual monomer and initiator coordination forms a reaction complex. The first step of the initiation stage is a nucleophilic attack with a formation of the tetrahedral carbon (or pentahedral phosphorus) followed by coordination of the exocyclic oxygen. The second step is a ring-opening, and the final step is a loss of the coordination of carbonyl (or phosphate) oxygen atom of the ring-opened product with simultaneous or subsequent coordination of the molecule of cyclic substrate at the metal atom. At the propagation stage, the alkoxy fragment plays the role of an initiator.

This “coordination-insertion” mechanistic concept was successfully applied in DFT modeling of ROP of different cyclic substrates catalyzed by the complexes of main-group, transition and rare-earth metals. Detailed DFT modeling of the ROP pathways for each particular case should take into account the following important issues:

- The qualitative difference in molecular structures of intermediates and transition states for initiation and propagation stages.
- Coherence and consistency of stationary points and transition states.
- The possibility of the specific coordination of the ring-opened product with a formation of stable chelate complexes.
- A variable coordination number for most metals.
- An option of the formation of bimetallic complexes and of the implementation of binuclear ROP mechanism.

Clarification and adjustment of the ROP mechanisms for each specific catalyst depends not only on the structure of metal complex but also on the nature of cyclic substrate. We think the second factor is more essential, and the material presented in this section of the review is organized by the monomer structure, i.e., lactones, cyclic carbonates, lactides, cyclic phosphates, and others. 

## 2. Coordination Polymerization of Lactones

As was stated earlier, the ROP thermodynamics plays a significant role in feasibility of the polymerization. In this regard this section is divided into subsections focusing on DFT modeling of ROP of highly strained lactones and moderately strained/non-strained lactones. 

### 2.1. Highly Strained Lactones

**Figure 2:**
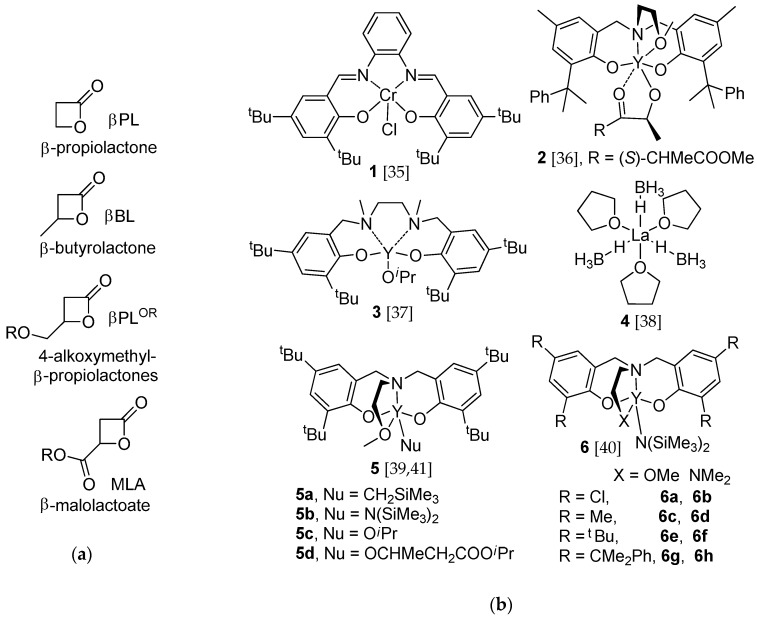
(**a**) Highly strained β-lactones; (**b**) Metal complexes studied in DFT modeling of β-lactones [35,36,37,38,39,40,41].

β-Lactones contain highly strained four-membered rings that lead to remarkable reactivity of these compounds in ROP. The parent compound, β-propiolactone, is of little practical interest, but the product of β-butyrolactone (βBL) ROP, poly(hydroxybutyrate) (PHB), is an attractive alternative to polyolefins owing to its mechanical properties comparable with the characteristics of isotactic polypropylene [33]. Highly isotactic PHB is a biosynthetic product, but controlled lowering of the isotacticity may improve the mechanical characteristics of PHB [34], therefore the stereoregularity of polymerization of substituted β-lactones is of crucial importance for the design of effective ROP catalysts. Only a few publications have focused on quantum-chemical modeling of polymerization of β-lactones (Figure 2a). The first study on the DFT modeling of βBL polymerization considered the chromium complex **1** [35], but the most of the catalysts studied are rare-earth metal complexes represented by compounds **2**–**6** [36,37,38,39,40,41,42,43] (Figure 2b).

The formation of isotactic PHB in β-butyrolactone polymerization, catalyzed by achiral chromium complex **1** (Figure 2b), was established experimentally and explained by the results of DFT modeling by Rieger et al. [35] at the BP86/SV(P) [44,45,46,47] level of theory using the TZVP basis set [48]. They demonstrated that the formation of isotactic PHB can be attributed to binuclear ROP mechanism via the formation of sandwich-like structure that opens a chiral reaction channel.

**Figure 3:**
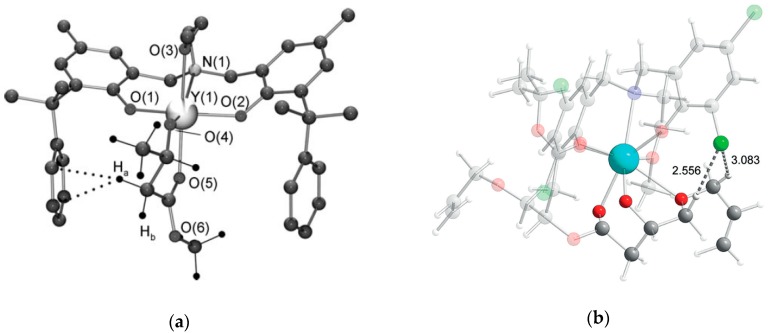
(**a**) DFT-optimized structure of the reaction intermediate with C–H···π interactions for βBL ROP catalyzed by **2**. Reprinted with permission from [36] Copyright (2011) Wiley-VCH Verlag GmbH & Co; (**b**) DFT-optimized structure of III-SS-TS based on **6a** and showing the shortest CH···Cl distances. Reprinted with permission from [40]. Copyright (2017) Wiley-VCH Verlag GmbH & Co.

The key findings on the mechanism of βBL polymerization, catalyzed by model yttrium complex **2** (Figure 2b), were made by Carpentier et al. [36] using DFT modeling at the BP86/def-TZVP level of theory [48,49]. They demonstrated that the valuable contribution of C–H···π interactions between the methylene hydrogen of the butyrate moiety (H_a_) and both the *ortho*- and *meta*-carbon atoms of a phenyl ring of CMe_2_Ph substituent (Figure 3a) control the formation of isotactic poly(βBL). The results reported in [36] were in line with the fundamental hypothesis of a ligand-assisted stereocontrol mechanism pointed out by Rzepa et al. [50] for lactide polymerization (see Section 4.1).

Thorough DFT modeling of *rac*-βBL polymerization catalyzed by yttrium complex **3** was performed by Thomas, Maron et al. [37] at the B3PW91 level of theory [51,52] in order to clarify the experimental fact of formation of syndiotactic polymer. The calculated activation barrier of the initiation stage was ~15 kcal/mol, and the chelate complex formed after the initiation stage was used of the model catalytic complex for the simulation of the propagation stage. Detailed analysis of eight reaction pathways based on the stereochemistry of (OCHMeCH_2_COO^i^Pr) chelate fragment and second molecule of βBL (*SS*, *SR*, *RS* and *RR* sequences), and chelate–monomer orientations (front-side and back-side) was performed. The results of calculations confirmed the preference of syndiotactic poly(βBL) formation without regard to the stereochemistry of the first inserted βBL molecule.

Polymerization of βBL catalyzed by borohydride complex [La(THF)_3_(BH_4_)_3_] (**4**, Figure 2b) was studied experimentally and theoretically at the B3PW91 [51,52] level of theory by Carpentier et al. [38]. It was demonstrated that at the initiation stage the complex anion BH_4_^−^ acts as a nucleophilic agent, BH_4_^−^ attack goes simultaneously with a coordination of exocyclic oxygen atom via the transition state with highest value of ΔG (14.5 kcal/mol). The initiation sequence involved the additional steps of hydride transfers with ring-opening leading to the formation of H_2_BOCH_2_CH_2_CH(O)Me alkoxy complex (oxygen atom in brackets is bonded with La atom). DFT modeling of the propagation stage was performed for two possible reaction pathways and resulted in conclusion about the equal possibilities of these mechanisms with the absence of stereocontrol. The formation of atactic polymer is in line with calculation results, but the reasons of sharp MWD (*Đ_M_* ~ 1.03–1.09) are unclear bearing in mind the multifaceted nature of the reaction mechanisms predicted *in silico*.

**Figure 4:**
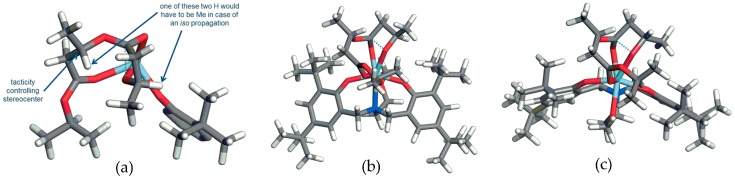
(**a**) Details within the structure of the lowest energy conformer of the rate limiting TS of βBL ring opening leading to the syndiotactic product and rationalization why this conformation disfavors an isotactic βBL incorporation; (**b**,**c**) Structures of lowest energy conformers of the rate-limiting TS of leading to syndiotactic (**b**) or isotactic (**c**) product. **5d** was used as a catalyst. Reprinted with permission from [39]. Copyright (2015) Wiley-VCH Verlag GmbH & Co.

Polymerization of βBL catalyzed by yttrium complex **5d** (Figure 2b) was studied by Rieger et al. [39] at the BP86/def-TZVP level of theory [44,45,46,47,48,49]. The full reaction profile was calculated; it was found that the formation of syndiotactic polymer is preferable due to the lower activation barrier (>5 kcal/mol). The detailed analysis of the stereochemistry of the conformers at the rate limiting transition states (Figure 4) allows one to explain this difference. 

**Figure 5:**
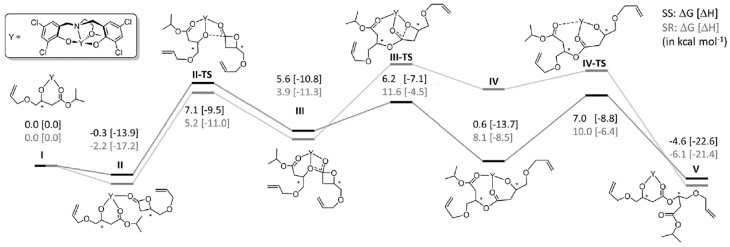
Energy profile of the proposed ROP mechanism for isotactic (black) and syndiotactic (gray) poly(βPL^OAllyl^) formation with **6a** as the initiator. Reprinted with permission from [40]. Copyright (2017) Wiley-VCH Verlag GmbH & Co.

Carpentier et al. studied ROP of 4-alkoxymethyl-β-propiolactones (βPL^OR^) catalyzed by a series of yttrium complexes with η^4^-coordinated NOXO-type ligands containing different substituents in benzene rings (**6a**–**h**, Figure 2b) [40] and found that the nature of substituents in benzene rings had a direct impact on polymer stereochemistry. At the same time, the nature of the alkoxy substituents in βPL^OR^ had no impact on stereoselectivity of ROP. DFT modeling of the second monomer insertion that is the key stage for explanation of the ROP stereoselectivity, was based on the results of previous work [37]. The calculated free energies (ΔG) of the first two steps for **6a**-catalyzed process were similar for both the iso- and syndioselective routes (Figure 5). However, the second transition state leading to syndiotactic poly(βPL^OAllyl^) (III-SR-TS) was notably higher in energy (ΔG^≠^ = 11.6 kcal/mol) than the transition state leading to an isotactic polymer (III-SS-TS, ΔG^≠^ = 6.2 kcal/mol). 

The origin was attributed to C–H···Cl interactions between the ring-opened monomer and the aromatic chloride substituents of the ligand (Figure 3b). As the free energy of III-SS-TS was lower, the actual rate-limiting step of the isoselective route was II-SS-TS with ΔG^≠^ = 7.1 kcal/mol. Hence, the activation energy for the formation of isotactic poly(βPL^OAllyl^) was 4.5 kcal/mol lower than for the syndiotactic polymer, which was in excellent agreement with the experimental result. The above calculations were repeated for methyl-substituted catalyst **6c** to estimate the differences for ligands with different substitution patterns. ROP of βPL^OAllyl^ using **6c** as the initiator proceeds through a similar route to that observed with **6a**, however, the free energies of almost all intermediates and transition states on the isotactic and syndiotactic propagation routes were virtually identical. This result was in perfect agreement with the experimental observation that the methyl-substituted catalyst **6c** produce atactic poly(βPL^OR^).

Luo et al. [41] performed DFT modeling at the B3LYP/6-31G*+ECP [51,53,54] level of theory of the initiation stage of βBL polymerization, catalyzed by yttrium 2-methoxyethylamino- bis(phenolate)s with different κ^1^-bonded ligands Nu (**5a**–**c**, Figure 2b) that act as nucleophilic initiators of ROP. The sequence of the SP and TS was close to the one presented in common Scheme 2. For Nu = ^i^Pr the relative free energy of the transition state of nucleophilic attack (TS-1) was minimal, but the ring-opening transition state TS-2 was higher by free energy. The calculated activation barriers of the initiation stage were 24.2, 22.6 and 19.0 kcal/mol for Nu = CH_2_SiMe_3_, N(SiMe_3_)_2_ and O^i^Pr, respectively.

Polymerization of benzyl β-malolactonate (MLA^Be^) catalyzed by rare-earth tris(borohydride) complexes was studied experimentally and theoretically by Maron et al. [42]. A comprehensive DFT modeling at the B3PW91 [51,52] level of theory with La(THF)_3_(BH_4_)_3_ (**4**, Figure 2b) as an initiator took into account the stereochemistry of nucleophilic attack and included the thorough analysis of initiation and propagation stages bearing in mind the possibility of –COOBn coordination and formation of chelates by ring-opened product. It was found that the value of the activation barrier of the initiation stage ΔG was 13.8 kcal/mol. The initiation stage resulted in H_2_BOCH_2_CH_2_CH(O)C(O)OBn chelate complex (oxygen atoms in brackets are bonded with La atom). It was demonstrated that two alternative pathways for coordination-insertion of the second monomer with comparable ΔG^≠^ values (~20 kcal/mol) are possible. The first pathway included the attack of BH_4_^−^, and the second pathway was an attack of coordinated alkoxy group formed at the initiation stage. The preference of the second pathway was elegantly confirmed by DFT simulations of the stereochemistry of the second MLA^Be^ insertion with a preferable formation of the syndiotactic polymer, which is consistent with experimental results.

The formation of syndiotactic polymers in ROP of allyl β-malolactonate catalyzed by yttrium complexes with η^4^-coordinated NOXO-type ligand with chloro substituents at benzene rings (**6a, b**, Figure 2b) was analyzed using DFT modeling by Carpentier et al. at the BP86-RI/def-TZVP level of theory [43]. They considered two possible types of the reaction mechanism, mononuclear and binuclear, taking into account the possible involvement of Cl···O halogen bonding between *ortho*-halogen substituents and oxygen from carbonyl/alkoxy groups of the propagating poly(alkoxybutyrate) chain. This bonding was detected in high-energy binuclear species. Thus, the mononuclear mechanism appeared to be more plausible, and the explanation of the syndiotacticity of polymerization of βBL derivatives was attributed to C–H···π(arene) interactions involving the acidic methylene hydrogen atoms of the coordinated *k*^2^-*O*,*O*-β-alkoxybutyrate fragment and the aryl rings in *ortho*-substituents CMe_2_Ph or CPh_3_ that was mentioned above [36].

Thus, ROP of strained lactones was studied using DFT modeling for numerous coordination catalysts. However, the results and, especially, methodology of these works that includes the analysis of the chelate formation, possibility of the binuclear mechanism, and clear understanding of the difference between initiation and propagation stages, are essential for DFT modeling of polymerization of other cyclic esters.

### 2.2. Moderately Strained and Non-Strained Lactones

**Figure 6:**
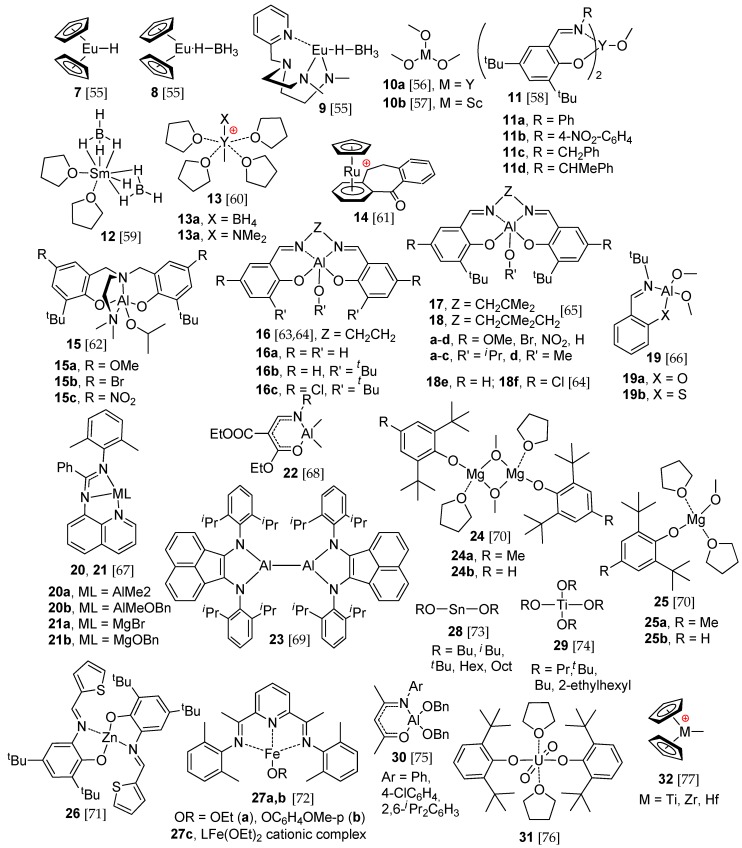
Metal complexes studied in DFT modeling of εCL ROP [55,56,57,58,59,60,61,62,63,64,65,66,67,68,69,70,71,72,73,74,75,76,77].

Most of publications on DFT modeling of the coordination ROP of lactones has addressed issues related to polymerization of ε-caprolactone (εCL, Scheme 1a) resulting in poly(εCL) that is an industrially important plastic. The use of metal catalysts for ring-opening polymerization of εCL have been thoroughly reviewed by Arbaoui and Redshaw [11]. With few exceptions, the fundamental principles of ROP of εCL may be extended to other lactones, cyclic carbonates and related compounds. Different metal complexes (Figure 6) have been studied in ROP of εCL using DFT methods. Generally, the mechanism of coordination polymerization of εCL is in line with the common ROP mechanism presented in Scheme 2. 

**Figure 7:**
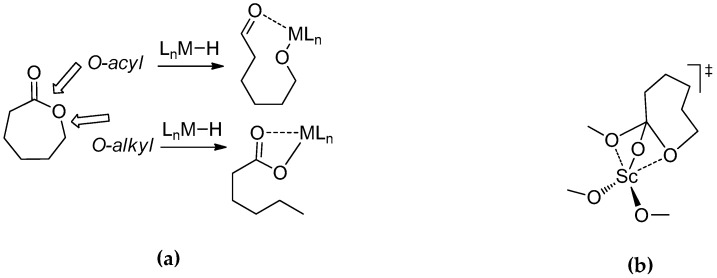
(**a**) Possible εCL ring-cleavage pathways in the reaction with metal-hydride complexes [55]; (**b**) The pendulum transition state [57] the likes of which are often overlooked in DFT modeling of ROP.

Maron et al. applied [(η^5^-C_5_H_5_)_2_EuH] model complex **7** for simulation of ROP of εCL, catalyzed by [(η^5^-C_5_Me_5_)_2_SmH]_2_ [55] using DFT method at the B3PW91/6–31G(d,p) level of theory [52]. The main problem to be solved was the determination of the preferred ring-cleavage pathway (Figure 7a). Thorough modeling allowed to draw conclusions on the preference of the O-acyl cleavage (the difference in free activation energies was more than 20 kcal/mol). The follow-up detailed comparative DFT modeling of the initiation of εCL polymerization was performed for **7**, borohydride complex **8** and post-metallocene borohydride complex **9** (Figure 6). The computational results were consistent with experimental studies for Sm complexes and, in particular, highlighted an important differences between hydride and borohydride initiation as well as between metallocene and non-metallocene ligand environment. The promise of the use of post-metallocene chelating ligands in design of rare-earth metal catalysts was highlighted, and it was the main result of this work, analyzing the retrospective of the coordination ROP of cyclic esters.

Shen et al. [56] investigated the mechanism of εCL insertion into a Y–OCH_3_ bond in complex **10a** (Figure 6) at the B3LYP level of theory [51,53] using the 6–31G* basis set for non-metal atoms and the LANL2DZ basis set [78] for yttrium. This study can be viewed as a scholarly work because the complex **10a** differs from the real ROP catalysts. The reaction sequence was fully in line with Scheme 2, but the authors have not optimized transition states of εCL insertion and ring-opening. A similar research was also carried out by Ling et al. [57] with scandium alkoxide **10b** (Figure 6). DFT calculations were performed at the B3LYP/6-31G* level [51,53,54] and resulted in the reaction sequence that differ substantially from Scheme 2. The most significant result reported in [56] was the finding of the “pendulum” transition state (Figure 7b) which is often neglected in DFT modeling of ROP of cyclic esters. DFT modeling of the geometry of ROP initiators **11a**–**c** was made Ni et al. [58] who made a useful conclusion about the correlations between Y–OMe bond length and catalyst activity.

Polymerization of εCL catalyzed by Sm (II) complex **12** (Figure 6) was studied by Maron et al. [59]. DFT modeling of concerted and oxidative reaction pathways resulted in estimation of the activation barriers by the values of 32.5 and 35.4 kcal/mol, respectively. The results of DFT modeling allowed them to propose that both processes will coexist in the reaction mixture.

The fundamentals of εCL ROP were re-investigated by Maron et al. using model cationic mixed-ligand Y complexes **13** (Figure 6) [60] at the B3PW91 [51,52] level using the 6-31G(d,p) basis set [79]; the Y atom was treated with a Stuttgart-Dresden relativistic pseudopotential [80]. The complexes under study included two combinations of Y-coordinated nucleophilic ligands, namely, BH_4_–Me and NMe_2_–Me. Four possible pathways of the nucleophilic attack to coordinated εCL molecule were estimated, the preference of O-acyl cleavage for all cases was demonstrated. It was found that the initiation of εCL ROP **13a** can occur on both the BH_4_ and Me ligand (ΔG^≠^ = 21.4 and 24.5 kcal/mol, respectively). The replacement of BH_4_ by NMe_2_ favored the reaction on the methyl side, indicating a trans effect. This finding was of great value for design of the novel coordination catalysts of ROP.

The bridge between homogeneous and heterogeneous catalysis was established by Maron et al. in a detailed study of the initiation of εCL ROP catalyzed by silica-grafted La complexes with different nucleophilic ligands, BH_4_, Me and NMe_2_ [81,82] at the B3PW91/6-31G(d,p) level [51,52,79]. It was demonstrated that silica-grafted complexes are able to initiate ROP, and that the reaction pathways leading to –CH_2_OBH_2_ or –CH_2_C(O)(X) (X = Me, NMe_2_) terminal groups seem to be kinetically and thermodynamically favorable. Me and NMe_2_ ligands initiated polymerization more effectively than BH_4_ (ΔG^≠^ 14–18 vs. 32 kcal/mol).

The ability of sandwich ruthenium complexes to catalyze εCL ROP was studied by Garcia et al. experimentally and theoretically [61]. DFT modeling at the B3LYP level [51,53] was performed for the stage of εCL coordination and initiation of polymerization by MeOH and complex **14** (Figure 6). Only one transition state was found for this process, the relative free energy of the TS was 41.9 kcal/mol. This high value of ΔG^≠^ correlate with very low activity of the catalyst. Note that the mechanism catalyzed by cationic complex **14** is markedly different from the conventional mechanism of living coordination polymerization by dissociation of the ring-opened product—metal complex.

The use of rare-earth metal complexes in catalytic ROP in the synthesis of biomedical-grade polymers might in practice be constrained quite considerably by their toxicity [83]. The complexes of biometals such as Al, Mg, Zn, Ca are highly attractive as ROP catalysts. The first theoretical studies of the mechanism of εCL ROP catalyzed by main-group metal complexes were performed in the early 2010s. Tolman et al. studied εCL polymerization, catalyzed by Al complexes **15** (Figure 4), supported by NONO ligands [62]. This work included the elements of DFT modeling at the M06-L [84] level of theory using the 6-31G(d) basis set for geometry optimization, and the M06-2X [85] level for thermochemical calculations. DFT modeling allowed to find a link between the electronic properties of the substituents in benzene rings of the ligands, and the ease of decoordination of NMe_2_ group that is necessary for ROP propagation. Different borohydride complexes of Ca were studied by Maron et al. [86]; these complexes demonstrated moderate activities, this experimental result correlated with DFT estimations of the activation barriers in the framework of the standard ROP mechanism, therefore these complexes are not discussed in this review.

**Figure 8:**
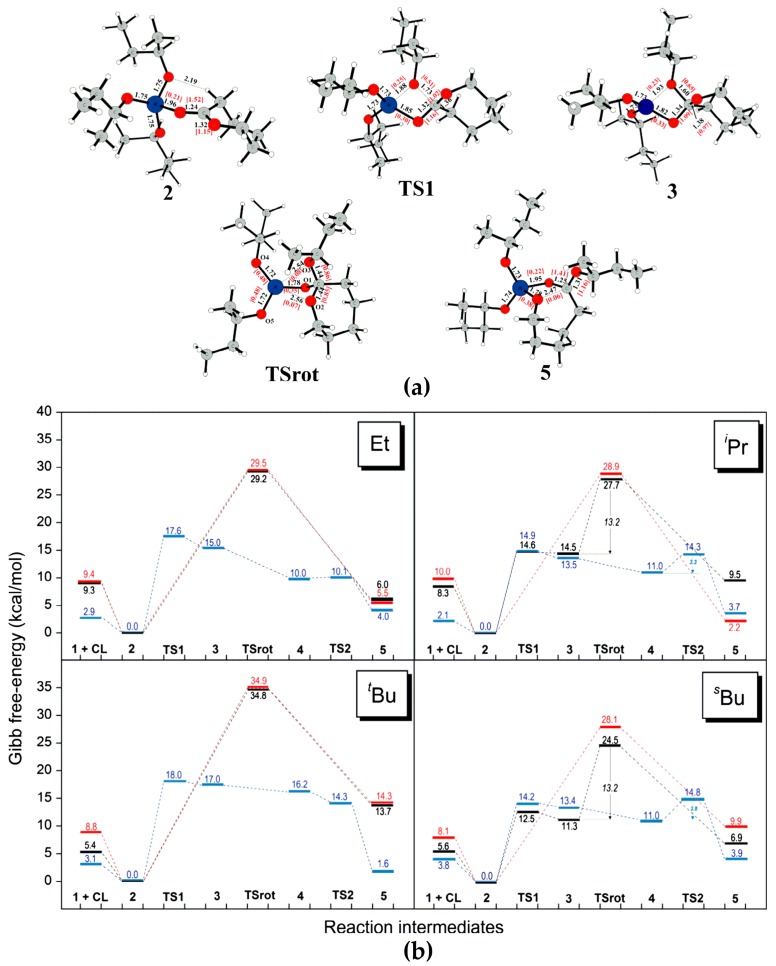
(**a**) Calculated structures of stationary points (**2**, **TS1**, **3**, **TS_rot_**, and **5**) along the εCL ROP pathway for Al(O^s^Bu)_3_ catalyst. Distances are given in Å. The Wiberg bond indices are indicated in brackets; (**b**) Calculated Gibbs free-energy profiles for the initiation of εCL ROP promoted by [Me_2_Al(OR)] (blue), [(MeO)_2_Al(OR)] (red) and [Al(OR)_3_] (black) with R = Et, ^i^Pr, ^t^Bu, ^s^Bu. Reprinted with permission from [87]. Copyright (2016) Elsevier B.V.

DFT modeling of εCL ROP catalyzed by truncated model complex **16a** (Figure 8) was performed by Tolman et al. [63] at the M06-L/6-31+G(d,p) [84]//M06-2X/6-311+G(d,p) [79,85] level. Thorough analysis of all possible coordination sites allowed to differentiate between “productive” and “non-productive” εCL coordination modes to explain experimental facts. The research was subsequently further developed with other Al complexes based on ONNO-type ligands, **17a**–**c** and **18a**–**c**. The simplified structures **17d** and **18d** (Figure 6) were used for DFT modeling of the key transition states [65] at the M06-L [84] level. Theoretical calculations demonstrated that ROP TS for **17d** and **18d** structures were very similar, thus raising the possibility that the differing activation energies for the two catalysts arise from differences in the energies required to distort the ligand framework to adopt the TS geometries. Approximating the energy cost of distorting the ligand framework from its reactant geometry to that of the TS for **16d** and **17d** through single-point calculations confirmed this conclusion. The relatively simple method for evaluating the ligand framework distortion energy proposed in [65] may have even broader utility for predicting the reactivity of metal alkoxide catalysts for cyclic ester ROP reactions and is therefore a potentially useful tool for future catalyst design.

Comprehensive and detailed modeling of εCL ROP, catalyzed by Al complexes **16** and **18e**,**f** (Figure 6) was performed by Chandanabodhi and Nanok [64] at the M06-2X/6-311G(d,p) level [79,85]. The sequence of SP and TS proposed in their work generally followed Scheme 2, and the calculations were performed for initiation and first propagation stages. The specific geometry of the ligand environment led to the absence of the pendulum TS. The calculated activation barriers decreased in the order **16b** (11.4 kcal/mol) > **18e** (7.4 kcal/mol) > **16c** (6.2 kcal/mol) > **18f** (5.8 kcal/mol), that illustrate the strong influence of the substituents in aromatic rings on catalytic activity.

The impact of alkoxy-groups in aluminium alkoxy and heteroleptic methyl-alkoxy complexes on the initiation stage of ROP of εCL was studied by Meelua et al. [87] at the B3LYP/6-31G*-LANL2DZ level [51,53,54,78]. DFT modeling of the reactions initiated by [Al(OR)_3_] and [Me_2_AlOR] complexes resulted in conclusion about the principal difference of the reaction pathways. for the Me_2_AlOR, the rate determining step of the initiation is the nucleophilic attack step **2**→**TS1**→**3** (E_a_ = 14.2–18.0 kcal/mol) but it is the ring-opening step (**3**→**TS_rot_**→**5**) for both [(MeO)_2_Al(OR)] and [Al(OR)_3_] systems which occurs through the penta-O-coordinated Al transition state, TS_rot_ (E_a_ = 28.1–34.9 kcal/mol and 24.5–34.8 kcal/mol, respectively, Figure 8). The finding of the rate-determining pendulum transition state TS_rot_ is an immense benefit of this work.

**Figure 9:**
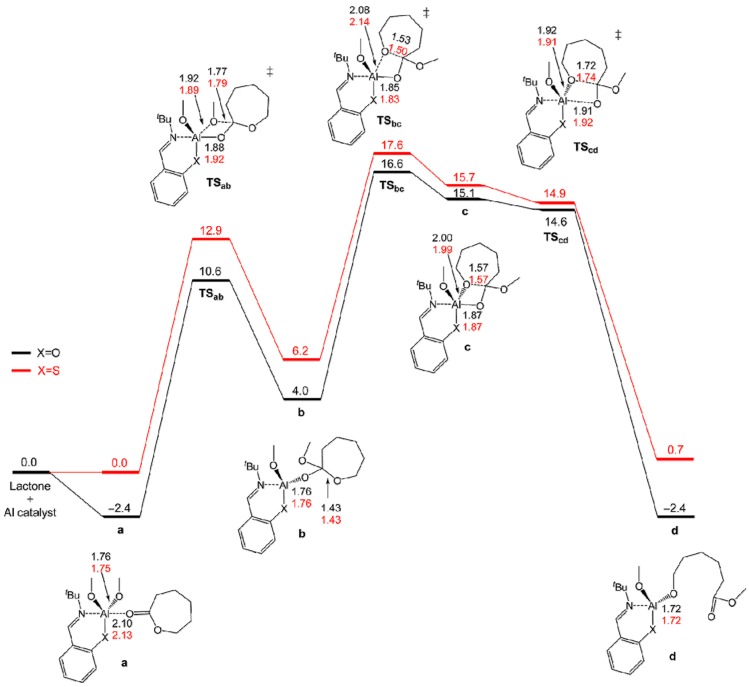
The free energy profile for ring-opening polymerization of εCL catalyzed by **19a** (black) and **19b** (red). The relative free energies (kcal/mol) and the selected bond lengths (Å) are given. Reprinted with permission from [66]. Copyright (2018) American Chemical Society.

Polydentate ligands mentioned above contained N and O donor atoms. Research groups at Taiwan Kaohsiung Medical University and National Sun Yat-Sen University studied experimentally and theoretically catalytic properties of Al complexes with aryloxy-imino and arylthio-imino ligands (**19a** and **19b**, respectively, Figure 6) [66]. The results of polymer-tests and DFT modeling at B3LYP-D3/6-311+G**//6-31G* level (Figure 9) demonstrated an obvious advantage of sulfur-containing complex **19b** as εCL ROP catalyst. It must be pointed out, that the pendilim transition state (the second TS in Figure 9) determines the activation barrier of the reaction. 

**Figure 10:**
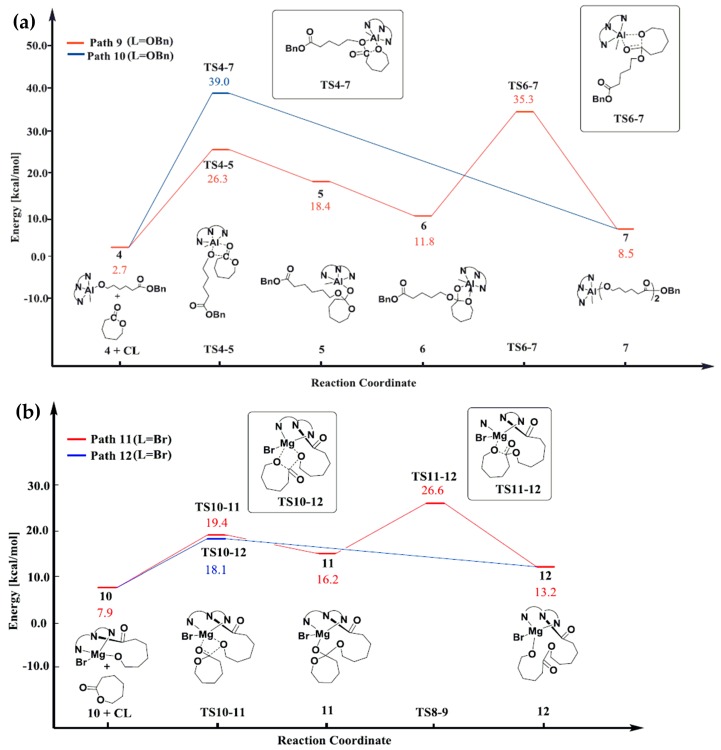
The Gibbs free energy profiles of the second molecule εCL insertion for ROP initiated by (**a**) Al complex **20b** and (**b**) Mg complex **21b**. Reprinted with permission from [67]. Copyright (2016) Elsevier B.V.

Al and Mg complexes (**20** and **21**, respectively, Figure 6) with specific NNN-type ligand were studied in εCL ROP by Sun, Lei et al. [67] using DFT modeling at the ωB97X-D/6-31G⁄* level of theory [79,88]. For Al complexes **20**, two different reaction mechanisms have been analyzed. The first of them was close to the mechanism presented in Scheme 2. The alternative way included the coordination of an exocyclic oxygen atom. At the initiation stage, the first way was found to be highly preferable, such preference has been maintained at the propagation stage (Figure 10a). In the case of Mg complexes **21**, one of the nitrogen atoms initiated ROP, and for **21b** the propagation resulted in formation of metallacyclic species (Figure 10b). In summary, the results of DFT modeling predicted higher catalytic activity for Mg complexes.

The influence of the geometry of catalytic complex based on aluminium chelate **22** (Figure 6) on activation barrier of εCL ROP was studied by Chen et al. [68] at the B3LYP/6-31G* level [51,53,54]. The nucleophilic attack to carbonyl carbon was found to be a limiting step of ROP, the value of the activation energy depended on the structure of the substituent at nitrogen atom of the chelate ligand, 4-chlorophenyl derivative demonstrated the best activity.

The use of chelating ligand allowed to obtain binuclear Al (II) complex **23** (Figure 6) with specific chemistry and catalytic properties [69]. It was found that **23** reacts with two equivalents of εCL with a formation of relatively stable adduct, its structure was confirmed by X-ray diffraction analysis. Compound **23** demonstrated a very poor activity in εCL polymerization, but after the addition of BnOH, the reaction rate increased by two orders of magnitude. DFT modeling of the initiation stage of εCL ROP at B3LYP/6-31+G** level of theory confirmed the binuclear character ot the reaction mechanism with explicit cooperative effect at the stages of the nucleophilic attack and ring-opening. 

**Figure 11:**
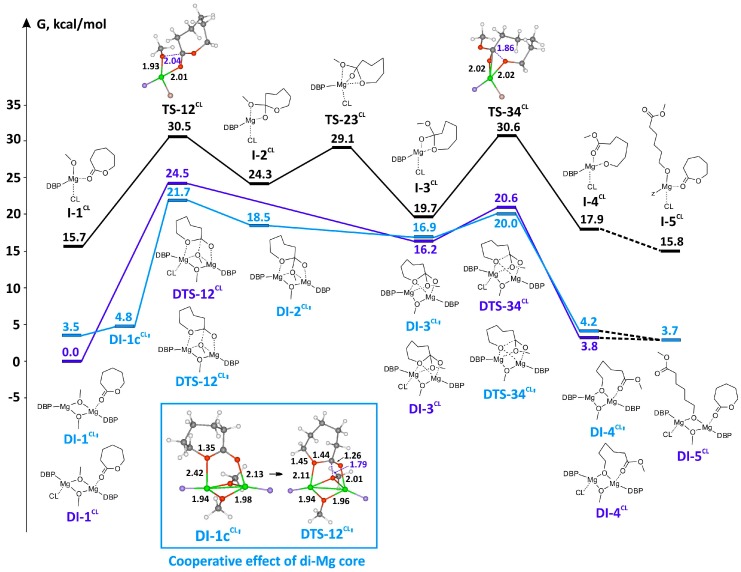
Reaction profiles of εCL ROP for binuclear and mononuclear coordination-insertion mechanisms (complexes **24b** and **25b**, respectively). DBP and second εCL fragments are omitted for clarity, phenolate and εCL oxygen atoms are colored in blue and pink. Reprinted with permission from [97]. Copyright (2017) Royal Society of Chemistry.

As noted above [67], Mg derivatives of the chelating NNN ligand outperform Al complexes in catalytic activity. In recent years, very simple and synthetically available aryloxy magnesium complexes drawn attention of the researchers due to their high catalytic activity in ROP [89,90,91,92,93,94,95]. In our study of Mg derivatives of 2,6-di-*tert*-butyl-4-methylphenol (BHT) we first proposed that the binuclear complex **24a** (Figure 6) dissociates with a formation of mononuclear species **25a [70]** and made DFT modeling of ROP of different cyclic esters using PRIRODA program [96]. Later, however, we studied the solution behaviour of BHT-Mg-OR complexes, confirmed binuclear nature of these species even in THF solutions, and performed a comprehensive analysis of εCL ROP mechanisms with the comparison of mononuclear and binuclear pathways [97] using DFT modeling with 2,6-di-*tert*-butylphenoxy (DBP) complexes **24b** and **25b** at B3PW91/DGTZVP level of theory [52,98]. The results of DFT simulations are presented in Figure 11. Note that our study was the first experience of the DFT modeling of binuclear coordination-insertion mechanism of lactone ROP, proposed earlier on the basis of experimental results [99]. The profiles obtained (Figure 11) demonstrated that the activation barriers of nucleophilic attack and ring-opening were equal for mononuclear mechanism, but the first TS was rate-limiting for binuclear mechanism due to facilitating of the ring-opening by cooperative effect caused by coordination of carbonyl and endocyclic oxygen atoms at two different Mg atoms.

**Figure 12:**
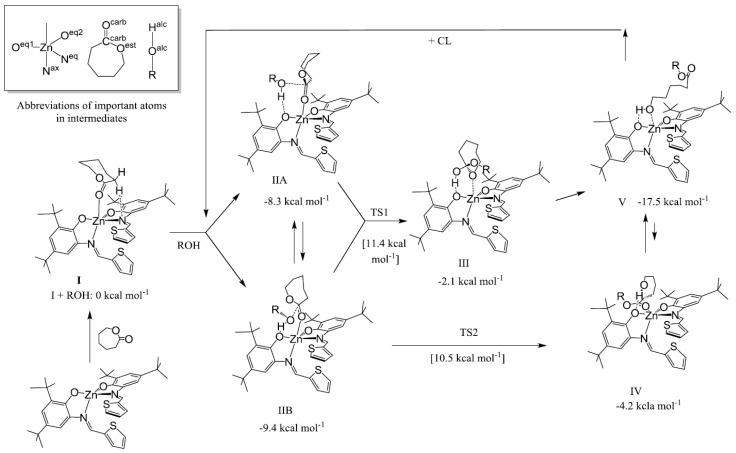
Proposed mechanism of εCL polymerization by using **26** as a catalyst. Reprinted with permission from [71]. Copyright (2019) Elsevier B.V.

Improved knowledge about coordination εCL ROP mechanism was obtained recently by Wu et al. [71] as a result of DFT modeling at the B3LYP/LANL2DZ/6–31g(d) [51,53,54,78] level of the process catalyzed by zinc complex **26** (Figure 6) and BnOH. The proposed mechanism involved the activation of εCL and BnOH molecules with substantial impact of the hydrogen bonding (Figure 12). 

Iron complexes typically do not demonstrate high catalytic activity in ROP of lactones. However, zero toxicity of iron-based catalysts has been a significant cause for the search of Fe-based polymerization catalysts. Recently, Cramer et al. studied εCL ROP catalyzed by bis(imino)pyridine Fe complexes **27** [72] with the use of DFT modeling at the M06-L level [84] Def2-TZVP and Def2-SVP basis sets (for Fe and residue atoms, respectively) [100]. It was found that high-spin species are more Lewis acidic than low-spin intermediates. The reaction profiles for initiation and propagation stages included three TS (insertion, pendulum and rate-limiting ring-opening).

Currently tin (II) salts are the most known metal-containing ROP catalysts due to the wide applications of Sn(Oct)_2_ (Oct–2-ethylhexanoate) in large-scale synthesis of polylactides and polylactones. Kungwan et al. used [73] DFT methods at the B3LYP/cc-pVTZ–LANL2DZ level [51,53,78] for the modeling of εCL polymerization catalyzed by Sn (II) alkoxides **28** (Figure 6). The sequence of the SP and TS used in modeling was the same as presented in Scheme 2. The barrier for ring-opening **TS-2** (Scheme 2) was the rate-limiting one, and relative energy values (19–26 kcal/mol) correlated with the bulkiness of the alkoxy groups. Note that the sums of electronic energies and ZPE were used to draw the reaction profiles, therefore correlations with experimentally determined activity of the catalysts seems to be ill-defined. Moreover, the very reason of such evaluations is unclear: it is amply evident that the structures of the alkoxy initiators after insertion and ring-opening of first few εCL molecules are almost identical. Thus, the similar estimations can clear the observations at the initiation stage (induction period etc.) but unable to explain the real catalytic activities of alkoxy complexes in living ROP. Exactly the same study of εCL ROP with Bu_3_SnOR (R = Me, Et, Pr and Bu) initiator were performed [101] two years following the first publication. For this complex, **TS-1** of the insertion step was the rate-limiting, and the methoxy initiator was found to be the most effective—as an *initiator*.

The critical points raised above hold true for other studies. Thus, the initiation of εCL ROP by Ti (IV) alkoxides [Ti(OR)_4_] (**29**, Figure 8) was modeled by Lawan et al. [74] at the B3LYP/LANL2DZ level [51,53,78] using a crude scan of the interatomic distances in Ti(OR)_4_–εCL complexes. The data obtained was correlated with the results of the polymerization tests. In our opinion, such a correlations are so flawed since the propagation rates should be determined by the insertion barriers in primary alkyl complexes ROC(O)(CH_2_)_5_OTi(OR)_3_ or their derivatives formed via εCL ring-opening ([ROC(O)(CH_2_)_5_O]_x_Ti(OR)_4–x_.

High-quality modeling of εCL ROP initiation by aluminium complexes **30** (Figure 8) was performed by Lin and Jheng [75] at the B3LYP/6–31g(d) [51,53,54] level using the polarizable continuum solvation model (PCM) [102]. They demonstrated that the step of benzoxide insertion was rate-limiting, and the bulkiness of aryl substituent in ketiminate ligand facilitated ROP. However, only initiation stage was studied thoroughly, and correlations between experimental data and calculated thermochemistry appeared controversial.

Other lactones, e.g., δ-valerolactone (δVL) or non-strained and non-polymerizable γ-butyrolactone (γBL) and γ-valerolactone (γVL) were investigated occasionally. In [87], Meelua et al. compared the reactivity of different ring-size lactones (εCL, δVL, γBL and γVL) in [Al(O^s^Bu)_3_]-catalyzed ROP. Similar to εCL, the initiation of ROP of other lactones proceeds via two transition states with the rate-determining second **TS_rot_**. The activation free-energies for the initiation were slightly increased with decreasing of the ring size (24.5, 27.3, 28.6, and 28.8 kcal/mol for εCL, δVL, γBL and γVL, respectively), thus, the polymerizability of 5-membered ring lactones (γBL and γVL) was kinetically less favorable.

A comparative DFT study of [Sn(OBu)_2_]-catalyzed ROP of γ-valerolactone (γVL), δVL and LA, was performed by Kungwan et al. [103] at the B3LYP/LANL2DZ level of theory [51,53,78]. The sequence of stationary complexes and TS applied in simulation of the reaction profile was fully in line with Scheme 2. Taking account of DFT modeling of ROP performed previously by this scientific group [73], the free activation energy values were 13.8, 18.6, 18.8 and 12.3 kcal/mol for γVL, δVL, εCL and LA, respectively. The authors estimated the activation energies as a difference between the free energy of the first transition state and the sum of the free energies of Sn(OBu)_2_ and corresponding cyclic ester, and proposed the following order of the rate constants: γVL = LA > εCL > δVL which is not in agreement with experimental observations.

**Figure 13:**
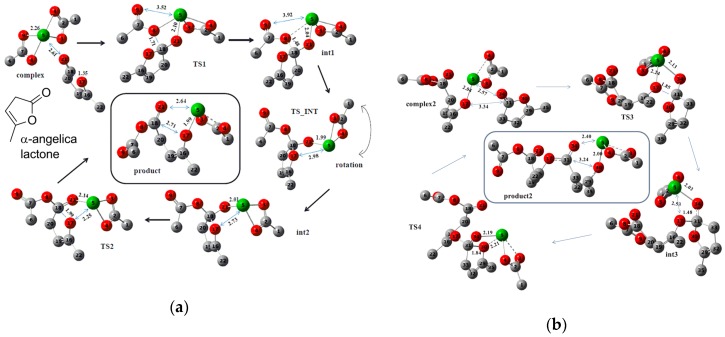
(**a**) Initiation and (**b**) propagation stages of the αAL polymerization catalyzed by [Sn(Oct)_2_]. Distances are given in Å. Oxygen, carbon and tin atoms are marked in red, gray and green, respectively. Reprinted with permission from [104]. Copyright (2011) Royal Society of Chemistry.

Five-membered lactones such as γBL have traditionally been considered as non-polymerizable due to the low strain energy of the ring and to the positive Gibbs free energy related to the polymerization [32]. However, the introduction of C=C bond into five-membered lactone results in monomer that can be polymerized by ROP. α-Angelica lactone (αAL, Figure 13) is an attractive cyclic substrate, furthermore it can easily be obtained from levulinic acid that is commercially available green bioplatform chemical. αAL has higher strain energy than γBL and negative enthalpy of polymerization although it is a five-membered lactone, this fact correlates with DFT modeling results [104]. Kınal et al. performed a theoretical study of αAL polymerization catalyzed by [Sn(Oct)_2_] by employing the semiempirical PM6, PM6-D3H4, PM7, and the DFT-B3LYP, B3LYP-D2 and ωB97X-D methods in both gas and solvent (toluene, THF, DMF and DMSO) media, Sn(II) acetate was used as a model initiator. In line with the results of the modeling, the initiation stage included the nucleophilic attack of carboxylate, weak coordination of endocyclic oxygen atom, and ring-opening (Figure 13a). The ring-opening stage was rate-limiting (ΔG^≠^ = 30–37 kcal/mol depending on DFT method used). The calculated activation barrier of the propagation stage (Figure 13b) was substantially lower (~15 kcal/mol). 

**Figure 14:**
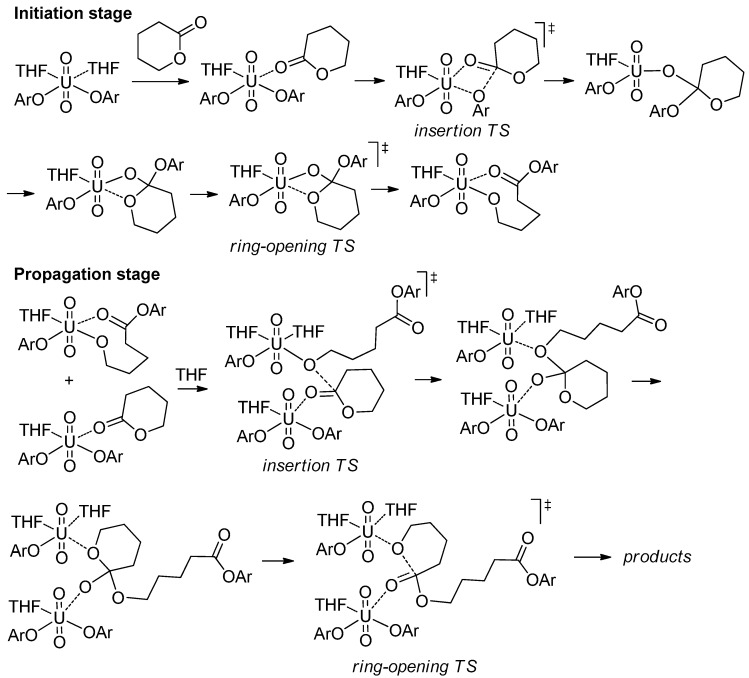
Intermolecular coordination-insertion mechanism of δVL ROP [76].

δVL and εCL ROP, catalyzed by uranium complex **31**, was studied experimentally and theoretically by Maron et al. [76]. The formation of macrocyclic polylactones was detected experimentally, the results of DFT modeling allowed to propose a novel type of ROP mechanism, namely, intermolecular coordination-insertion involving two uranyl catalytic species (Figure 14). The calculated free activation energies for the stage of the insertion and ring-opening of the second lactone molecule were 14.0 and 20.8 kcal/mol for δVL and εCL, respectively. The authors proposed that a number of mechanism studies of lactone ROP may have to be reinvestigated to consider intermolecular pathways, and this assumption feels right and prospective for further study of polymerization of cyclic esters.

**Figure 15:**
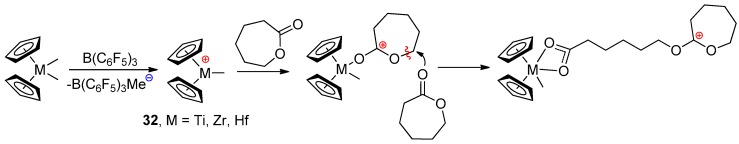
Generic mechanism for the cationic ROP of lactones using **32** as a catalysts [77].

Except cationic complex **14**, the initiators mentioned above catalyze ROP of lactones by similar mechanisms that include nucleophilic attack of the metal-coordinated ligand to carbonyl group followed by ring-opening via O-acyl bond cleavage (Scheme 2). The mechanism of ROP catalyzed by **14** slightly differ from this common scheme by the participance of non-coordinated nucleophilic agent (ROH). However, metal-catalyzed ROP of lactones may occur by a far different living cationic mechanism (Figure 15) that was studied in silico by Jitonnom and Meelua for (η^5^-C_5_H_5_)_2_MMe cations **32** [77] at the B3LYP/6-31G*-LANL2DZ [51,53,54,78] level. These calculations were performed for gas phase and using CPCM solvent model for THF, CHCl_3_, toluene and hexane. The reactivity of other lactones and cyclic carbonates was also estimated, taking into consideration the donor properties of monomers and the ring strain. Technically, this reaction cannot be regarded as coordination-insertion ROP, but the nature of the complex **32** have a direct impact on the initiation step, therefore we included this work in the review. 

## 3. Coordination Polymerization of Cyclic Carbonates

A number of publications devoted to DFT modeling of ROP of lactones take into account the prochirality of lactones which increases the calculation time and complicates the analysis of the reaction profiles. Cyclic carbonates, e.g., trimethylene carbonate (TMC, Scheme 1a), are more convenient substrates with regard to the stereochemistry of ROP. Cyclic carbonates are of lesser interest in terms of practical use of the ROP products, but DFT modeling of the polymerization of cyclic carbonates is of general importance for theoretical study of the polymerization of cyclic esters.

The first DFT modeling of TMC ROP was made by Ling et al. [57] with scandium alkoxide **10b** (Figure 6). The calculations predicted similar reactions rates for εCL and TMC by the values of the rate-limiting pendulum transition states (shown at Figure 7b for εCL). The similar study was carried out by Ling et al. for 1-methyltrimethylene carbonate (MTMC) and TMC ROP using Y(OMe)_3_ (**10a**, Figure 6) as a catalyst [105] at the B3LYP/LANL2DZ + 6-31G* level. Only insertion TS were found, and the authors estimated the free activation energies by the values of 9.3 and 9.5 kcal/mol for TMC and MTMC, respectively, as a difference between G(TS) and the sum of the free energies of Y(OMe)_3_ and cyclic carbonates. We cannot agree with this assessment, and we think that the activation barrier of ROP should be calculated as a differences between the free energies of TS and ground states (GS, for the topic at hand GS represent the complexes of Y(OMe)_3_ with TMC or MTMC). Such correction resulted in ΔG^≠^ values of 19.5 and 20.2 kcal/mol.

**Figure 16:**
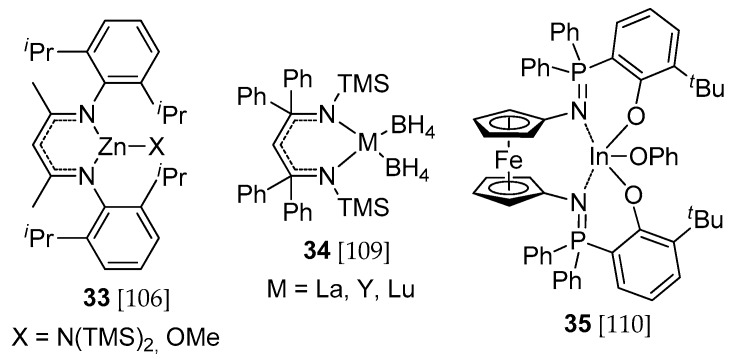
Metal complexes studied in DFT modeling of cyclic carbonates [106,109,110].

Maron et al. performed detailed and comprehensive modeling of TMC and tetramethylene carbonate (7CC) ROP catalyzed by Zn diketiminates with N(TMS)_2_ and OMe nucleophilic ligands **33** (Figure 16) [106] at the B3PW91 [51,52] level using Stuttgart effective core potentials and their associated basis set [107] for zinc and 6-31G(d,p) [79] basis set for other atoms. N(TMS)_2_ was a poor initiator (ΔG^≠^ ~ 50 kcal/mol), but the relative values of the rate-limiting first TS of insertion with OMe initiator were 17.4 and 16.1 kcal/mol for TMC and 7CC, respectively. The activation barriers of the propagation stage were calculated as 20.8 and 18.0 kcal/mol for TMC and 7CC, these barriers also respond to relative free energies of the insertion transition states. It was significant that the reaction pathways of ROP of carbonates catalyzed by **33** did not included the pendulum transition states. The research was subsequently further developed for γ-substituted six- and seven-membered cyclic carbonates [108], DFT modeling data were in good agreement with the experimental results including stereochemistry of ROP.

**Figure 17:**
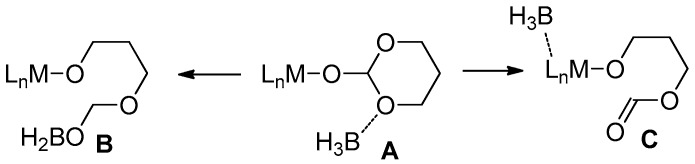
Unique intermediate and two possible reaction pathways for TMC ROP catalyzed by complexes **34** [109].

DFT modeling of TMC ROP initiation by rare-earth metal borohydride complexes **34** was performed by Guillaume et al. [109] at the B3PW91 [51,52] level. Several unprecedented features were revealed in comparison with the initiation of lactone ROP [60,81,82]: the formation of easy accessible (without activation barrier) intermediate **A** after rate-limiting nucleophilic attack of BH_4_ to TMC molecule (ΔG^≠^ ~ 24 kcal/mol) was located, and two distinct processes with similar activation barriers have been computationally identified. These processes resulted in qualitatively different reaction products **B** and **C** (Figure 17).

**Figure 18:**
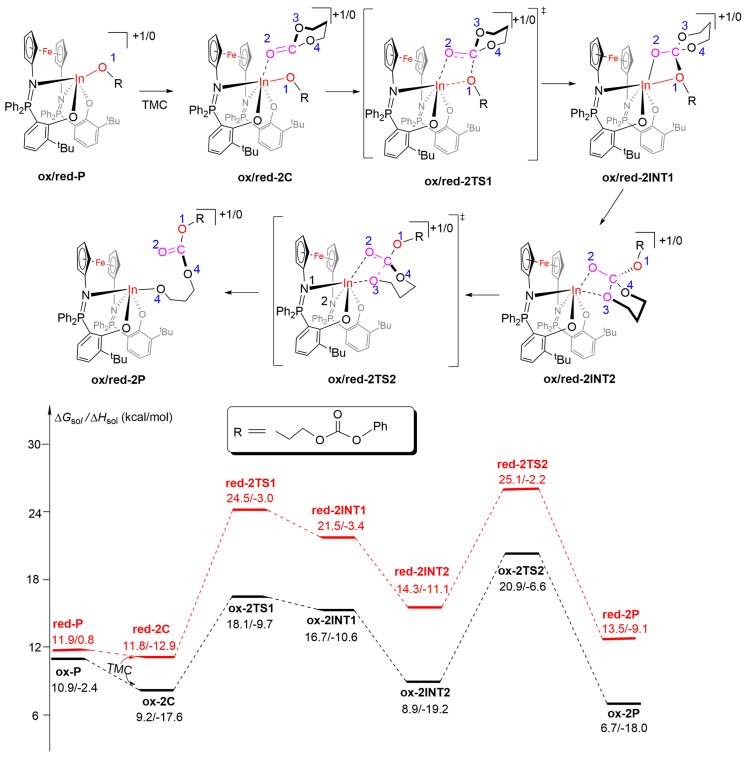
Computed energy profiles for chain propagation step of ROP of TMC catalyzed by cationic and neutral (Fe^III^/Fe^II^) indium complex **35**. Reprinted with permission from [110]. Copyright (2018) American Chemical Society.

The use of heterobimetallic complexes is one promising strategy in catalyst design [111,112]. In TMC ROP, indium complex **35** bearing a with redox-switchable ferrocene-based ligand was studied experimentally and theoretically [110] at B3LYP/6-31G* [51,53,54] level (Figure 18).

The calculations demonstrated that ring-opening was the rate limiting step and indicated that the oxidized state of the complex **35** had higher activity than the reduced analog. The results of modeling were in good agreement with experimental observations.

Cationic coordination polymerization of TMC catalyzed by different zirconocenes was studied in silico by Jitonnom and Meelua [113,114] using DFT calculations at the B3LYP/6-31G(d)-LANL2DZ [51,53,54,78] level of theory. The reaction mechanism was similar to the mechanism presented in Figure 15. The main idea of this work was to find correlation between the structures of zirconocene precatalysts and activation barriers of ROP. A representative sample of 29 zirconocenes was studied, and it was demonstrated that the steric and electronic properties of the zirconocene catalyst can be tuned by ligand modifications. Note that the results of these works are important for other zirconocene-catalyzed processes such as α-olefin polymerization since the reactivity of cationic complexes formed after the first TMC insertion can be seen as a measure of the electrophilicity of zirconium cationic catalytic center. Finally, it can be mentioned that the results of DFT modeling of TMC ROP were reported in some publications mentioned above [70,77].

## 4. Coordination Polymerization of Lactides and Glycolide

### 4.1. Monomuclear ROP Mechanism

**Figure 19:**
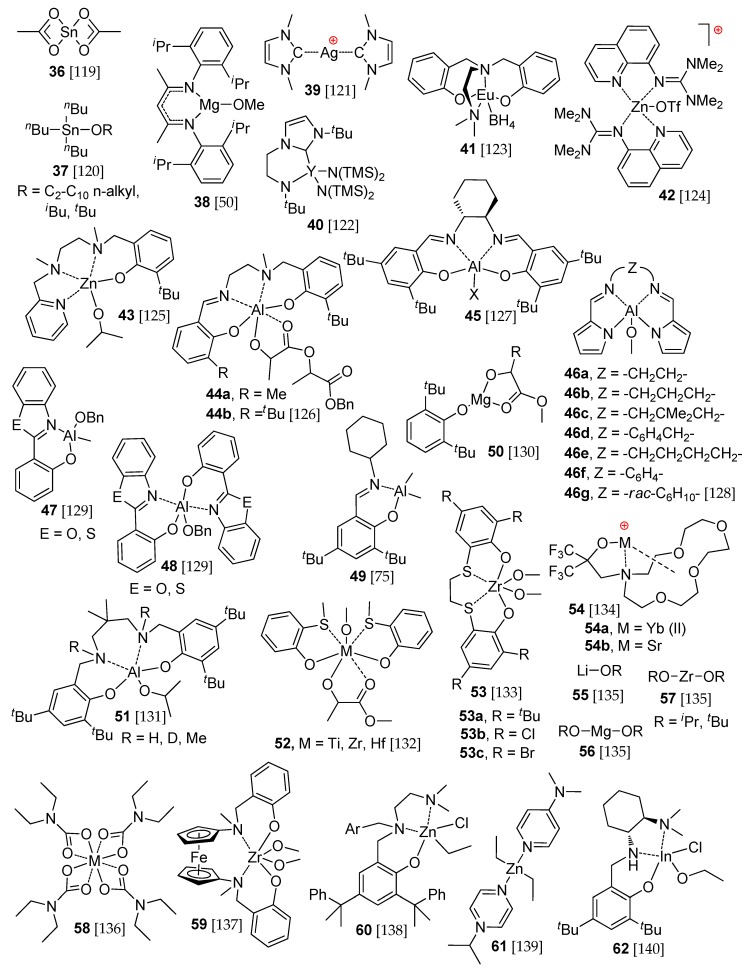
Mononuclear metal complexes studied in DFT modeling of lactide ROP [50,75,119,120,121,122,123,124,125,126,127,128,129,130,131,132,133,134,135,136,137,138,139,140].

Coordination ROP of lactide (LA) is of great practical importance in the light of biomedical applications of polylactic acid (PLA) and related polymers [115,116,117,118]. The mechanisms of coordination LA ROP are by far the most detailed, DFT modeling was effectively used for a better understanding of kinetics and stereochemistry of polymerization, catalyzed by different metal complexes (Figure 19). 

The stereochemistry of the starting cyclic monomers must be taken into account when analyzing the reaction mechanisms and polymer microstructure: l-lactide (*l*LA) and (dl)-lactide ((dl)LA, 1:1 racemic mixture of l- and d-isomers) frequently demonstrate different reactivities due to steric effects arising in the catalytic complex comprised of the metal atom, coordinated LA molecule and growing PLA chain.

**Figure 20:**
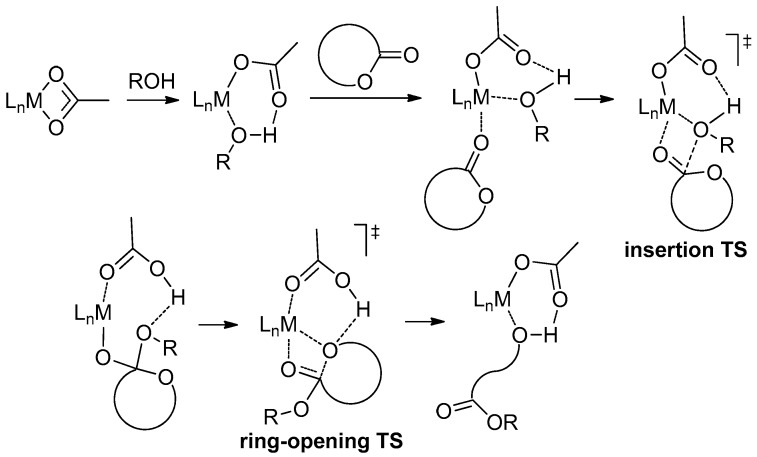
Common mechanism of the coordination ROP of cyclic esters catalyzed by metal carboxylate and alcohol [119].

LA ROP was studied using DFT modeling before the rest of other cyclic esters. In 2001, Svensson et al. performed DFT calculations for l-LA ROP, catalyzed by [Sn(OAc)_2_] (**36**, Figure 19) with MeOH initiator [119] at the B3LYP/LANL2DZ level of theory [51,53,78]. We do not entirely agree with optimized geometry of l-LA, reported in [119], our calculations clearly predicted the preference of boat-like conformation with double equatorial orientation of the methyl groups [97,130,141]. Nevertheless, the results of the modeling allowed to make important conclusions about the mechanism of ROP catalyzed by metal carboxylates as follows: the endergonic formation of the complexes with alcohols, and essential assistance towards the nucleophilic attack and ring-opening by hydrogen bonding (Figure 20). The activation barrier for l-LA ROP was determined to be 20.6 kcal/mol. So low value of ΔG^≠^ seems to be contrary to moderate catalytic activity of tin (II) carboxylates in LA ROP. Thirteen years later, Kungwan et al. [103] carried out DFT modeling of [Sn(OBu)_2_]-catalyzed *l*LA ROP at the B3LYP/LANL2DZ level of theory [51,53,78]. The calculated value of the activation barrier was 12.3 kcal/mol, this result is also appears not quite correct bearing in mind moderate catalytic activity of Sn (II) alkoxides in lactide polymerization. One would expect that the cause of such potential underestimation of the activation energy can be explained by imperfection of the model used. [Sn(OBu)_2_]-based catalytic species were presented in [103] as complexes having CN = 2 or 3 (even for TS). Clearly, such species contain highly electrophilic Sn center, which accelerate insertion and ring-opening of LA, but very plausible coordination of additional LA molecule was ignored.

**Figure 21:**
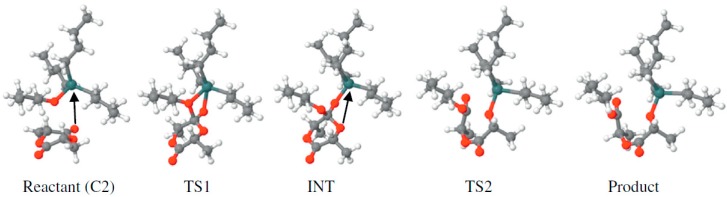
Molecular structures of SP and TS involved in ROP of *d*-lactide with **37** (R = Et). Arrows are drawn to emphasize moving directions of oxygen atoms. Reprinted with permission from [120]. Copyright (2013) Elsevier B.V.

DFT modeling of d-LA ROP catalyzed by ^n^Bu_3_Sn–OR **37** (Figure 19) was performed by Lawan et al. [120] at the B3LYP/LANL2DZ [51,53,78] level. The optimized molecular structures of SP and TS for **37** (R = Et) are presented in Figure 21. Evidently, these structures are directly relevant to the initiation stage of ROP. However, the reliability of the method used to find the TS, namely, the direct scanning by the energy with variable distances as pointed by arrows in Figure 21 without saddle point optimization and IRC test, is debatable.

**Figure 22:**
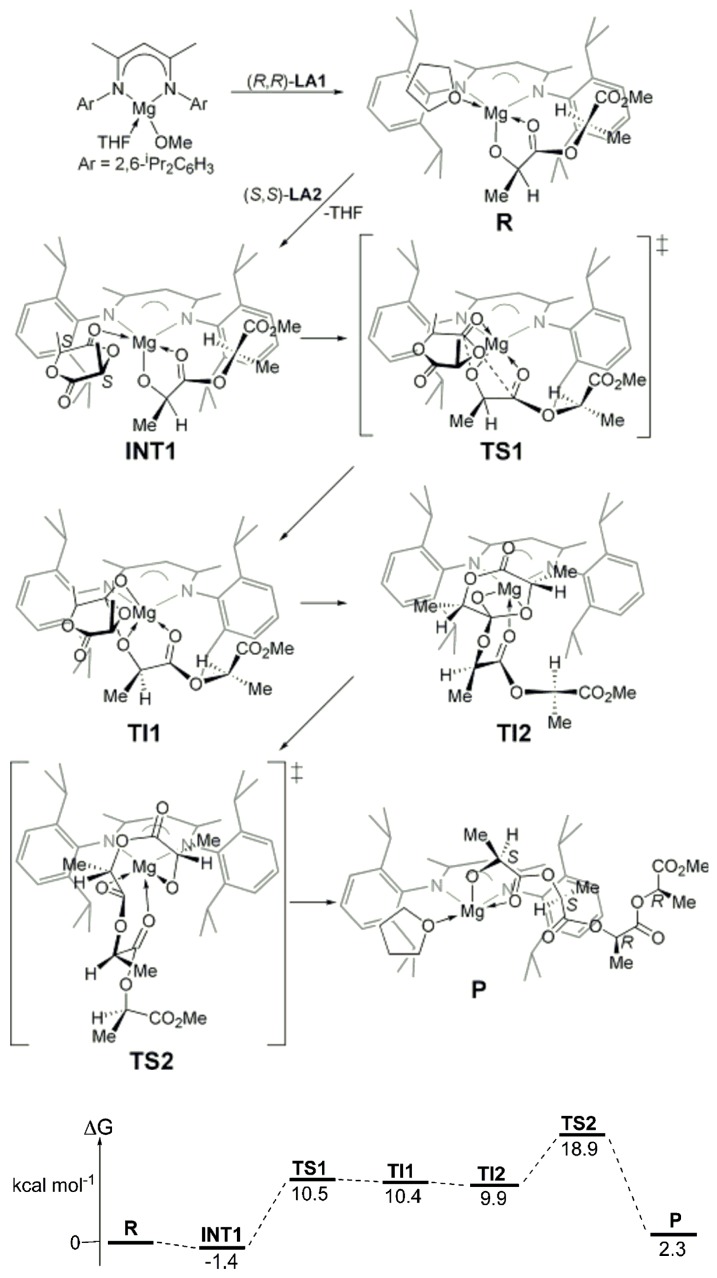
The structures of the catalytic species and calculated free energy profile corresponding to the RR,SS assembly mode of *dl*LA ROP catalyzed by **38**. Reprinted with permission from [50]. Copyright (2005) American Chemical Society.

It is perhaps not an exaggeration to declare that the detailed investigation of (dl)-LA ROP done by Rzepa et al. [50] was of fundamental importance for the theory and practice of coordination polymerization of cyclic esters. The authors optimized key intermediates and transition states for every possible configurations of chelating lactate fragment and coordinates LA molecule, and found the factors influencing stereoselectivity at a magnesium center in catalytic species formed from the complex **38** (Figure 19). For this complex, the rate-limiting step was the ring-opening of LA molecule, and steric interactions in corresponding transition state determined the stereoselectivity of ROP with a formation of heterotactic PLA in (dl)-LA ROP (Figure 22).

Despite the simplicity of the scandium initiator used (**10b**, Figure 6), Ling et al. clearly demonstrated all key stationary points and TS of l-LA ROP [57]. The apparent energy barriers of the initiation stage for εCL, TMC and l-LA were calculated as 22.3, 20.7 and 17.0 kcal/mol, respectively. However, the tetrahedral model proposed for Sc-mediated polymerization appears to be incomplete due to neglect of the coordination of additional l-LA molecules. 

**Figure 23:**
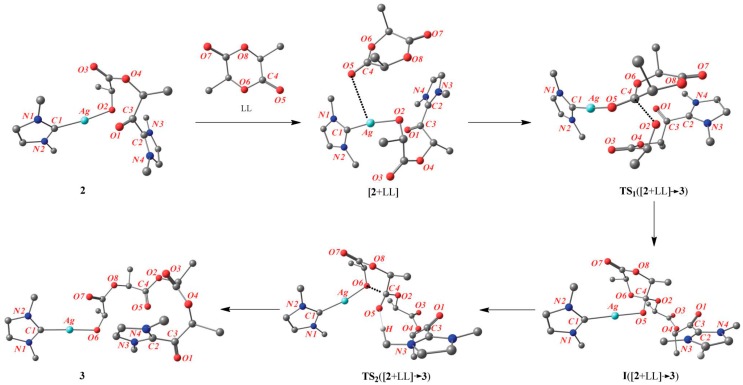
Propagation step of the l-lactide polymerization by **39**. Reprinted with permission from [121]. Copyright (2011) Royal Society of Chemistry.

The mechanism of LA ROP catalyzed by N-heterocyclic carbene (NHC) silver (I) complex **39** (Figure 19) was studied by Ghosh et al. [121] at the B3LYP/6-31G(d) level of theory [51,53,54]. This complex demonstrated a non-trivial behaviour at the initiation step, the NHC molecule acted as a ROP initiator with a formation of alkoxy complex that has been regarded as a chain propagation particle (Figure 23). The free activation barrier for propagation stage was estimated to have a value of 31.5 kcal/mol.

**Figure 24:**
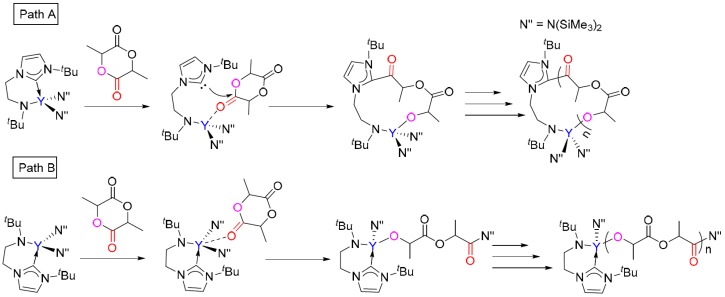
Possible reaction pathways for LA ROP catalyzed by complex **40** [122].

A significantly more challenging reaction mechanism was proposed and analyzed by DFT modeling of (dl)-LA ROP catalyzed by yttrium complex **37** with constrained-geometry NHC ligand [122]. At the initiation stage, both NHC and N(SiMe_3_)_2_ fragments can act as nucleophilic agents with a formation of macrocyclic and linear dilactate complexes, respectively (Figure 24). The third possible way included the loss of Y-NHC coordination, was excluded by the results of DFT modeling at the B3PW91/6-31G(d)-LANL2DZ [51,52,54,78] level. From the calculation results, the pathway B was considered as favorable. The modeling of the propagation stage (insertion of the second LA molecule) allowed to make a conclusion about the preference of l-LA insertion after the first d-LA insertion and ring-opening that correlated with the formation of heterotactic PLA observed experimentally.

A theoretical study of (dl)-LA ROP catalyzed by Eu borohydride complex **41** (Figure 19) [123] was performed at the B3PW91 [51,52] level using the 6-31G(d,p) basis set [79]; the Eu atom was treated with a Stuttgart-Dresden relativistic pseudopotential [80]. The results of modeling shown that the reaction pathway leading to a –CH(Me)–CH_2_OBH_2_-terminated growing chain was kinetically accessible and thermodynamically favorable. The initial hydride transfer step from Eu-BH_4_ to a lactide C=O carbon and partial transfer of the residual BH_3_ to the exocyclic oxygen was reminiscent of the mechanisms for [(l)Ln(BH_4_)]-catalyzed εCL ROP [55,59,60]. However, a substantial difference between [(l)Ln(BH_4_)]-catalyzed polymerization of lactones and lactides was pointed out. For LA, the activation of ROP included two steps, namely, the formation of ring-opened intermediate with a BH_3_-capped chelating aldehyde group –CH(Me)CHO·BH_3_ (first step) and thermodynamically more likely second irreversible hydride transfer from BH_3_ to the –CH(Me)CHO carbonyl carbon with α,ω-dihydroxy poly(dl-LA) formation (second step). The results of modeling were consistent with the results of polymerization experiments.

High significance of the formation of five-membered metallacycle in LA ROP had been demonstrated by Herres-Pawlis et al. [124] by the case of zinc guanidine complex **42** (Figure 19). Detailed experimental study and DFT modeling at the B3LYP/6-31G(D) and 6-311G+(d) levels [51,53,54,79] resulted in estimation of activation barriers of the propagation stage as 19 and 15.5 kcal/mol, respectively. The ability of **42** to promote LA ROP without additional alkoxy initiators was confirmed by DFT modeling of the initiation stage. Low activation barrier value of the initiation stage (24 kcal/mol) make it possible to assess **42** as a prospective single-component ROP catalyst. Zn complex **43** based on specific NNNO ligand (Figure 19) demonstrated excellent catalytic activity in lactide polymerization [125]. DFT modeling of the initiation stage at the M06-L [84] level using the 6-31+G(d,p) [79] and the SDD [107] basis set resulted in estimation of the activation barrier (ring-opening TS) by the value of 12.4 kcal/mol that was in good agreement with high activity of the catalyst. 

**Figure 25:**
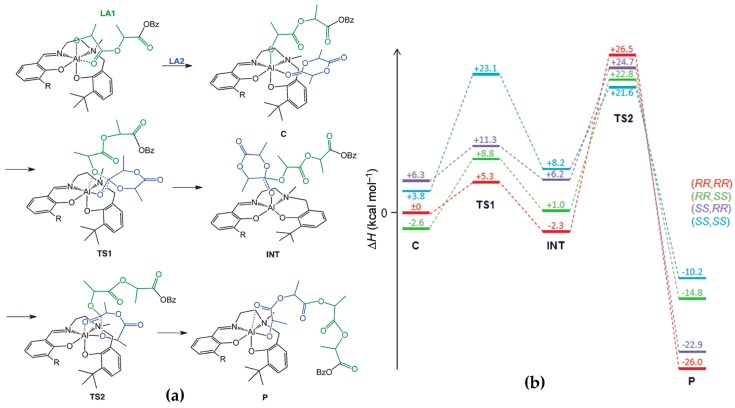
(**a**) Mechanism for the chain growth in ROP of LA with Al complexes **44**; (**b**) Calculated enthalpies (kcal/mol) for the reaction coordinates of the chain-propagation model with complex **44a**. Reprinted with permission from [126]. Copyright (2013) Wiley-VCH Verlag GmbH & Co.

ONNO-ligated Al complexes **44** (Figure 21) were studied as (dl)-LA ROP catalysts by Jones et al. [126] experimentally and theoretically at the B3LYP/6-31g(d) level [51,53,54]. This short communication presented a detailed analysis of the causes of heterotactic and atactic PLA formation for catalysts **44a** and **44b**, respectively, from the mechanistic point of view (Figure 25). The effective control of (dl)-LA ROP stereochemistry by minimal changes in ligand structure (see Figure 25, R = Me or *^t^*Bu—heterotactic or atactic PLA) should be regarded as an effective tool in design of prospective ROP catalysts.

Further study of Al catalysts with ONNO ligands **45** was performed by Maron, Thomas et al. [127] in (dl)-LA ROP initiated by bis(triphenylphosphoranylidene)iminium) chloride in propylene oxide media. DFT modeling predicted the formation of anionic catalytic species containing two alkoxy fragments and, therefore, possibility of bifacial chain propagation. The mechanism of LA ROP predicted by DFT modeling was substantially different from the common ROP mechanism (Scheme 2) by the ease of the release of anionic alkoxy group during LA coordination. Both initiation and propagation stages were thoroughly analyzed and discussed, the absence of chelate formation due to specific ligand enviroment is the likely reason for extremely high activity of this catalytic system in lactide polymerization.

Hormnirun et al. [128] performed DFT modeling of (dl)-LA ROP catalyzed by Al complexes **46** (Figure 19) with different alkylene bridges between pyrrole rings at the M06-2X/6-311G(d,p) [79,85] level of theory. It is worth highlighting the geometries of the reaction intermediates and TS which were defined by the nature of the bridge Z, and the direct impact of chelate formation into stabilization of the products of the initiation and propagation stages. The comparative study of the insertion of d-LA or l-LA into the complex formed by first enchained l-LA monomer unit allowed to explain the stereochemical results of the polymerization experiments.

Initiation stage of (*dl*)LA ROP catalyzed by aluminium benzo[*d*]oxazole and benzo[*d*]thiazole complexes **47** and **48** was studied by Hormnirun et al. [129] at the M06-2X/6-311G(d,p) level [79,85]. In accordance with the DFT modeling results, the initiation stage ends with a formation of chelate complexes; this process has been accompanied by lowering of the free energy (7.7–12.1 kcal/mol). The lack of DFT calculation data for the propagation stage reduces the value of the study with regard to correlations with experimental data on catalytic activity of **47** and **48**.

The results of the modeling of LA ROP initiation by aluminium complexes **49** (Figure 6) performed by Lin and Jheng [75] demonstrated that the step of the ring-opening was rate-limiting. Note that this pattern is common for LA ROP catalyzed by metal complexes of different structures.

The catalysts mentioned above represent either simple alkoxides or complexes with polydentate ligands. The mechanisms of the LA ROP initiated by catalysts with interim complexity and synthetic availability until recently were not studied using DFT modeling, except moderately active complex **39**. The use of main-group metal complexes with bulky aryloxy ligands is highly promising in ROP, and recently we studied a number of heteroleptic [BHT-Mg-OR] complexes in εCL ROP [70,93] (see Section 2.2) and LA ROP. Based on assumption about mononuclear nature of the catalytic species [93], we preformed DFT modeling of (dl)-LA ROP catalyzed by complexes **50** (Figure 19) containing one or two monomer molecules coordinated at Mg atom in [130]. The modeling was performed at the PBE/3ζ level [142], the preferable CN value (4, one monomer molecule coordinated) was determined by draft simulations of glycolide ROP, and the geometries obtained were used in (dl)-LA ROP modeling. To explain experimental fact of heterotactic PLA formation, we studied all possible combinations of coordinated methyl lactate and lactide taking into account the stereochemistry of CHMe fragments, and established a notable difference in free activation energies (>2 kcal/mol) for heterotactic and isotactic PLA formation. However, subsequently, the results of this study were revised in the light of binuclear ROP mechanism [97] (see Section 4.2).

**Figure 26:**
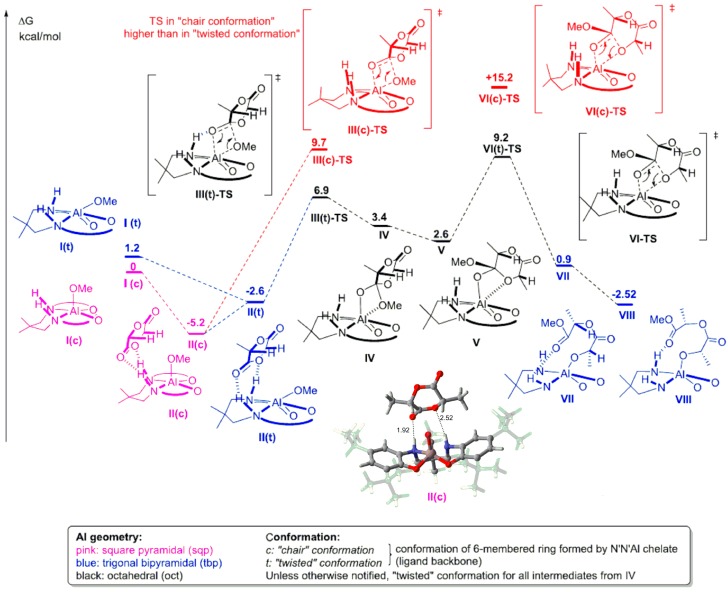
Potential energy surface corresponding to first *l*LA insertion (initiation step of (dl)LA polymerization catalyzed by **51** and molecular structure of the intermediate II(c) showing docking of the *l*LA on the top of the initiator. Reprinted with permission from [131]. Copyright (2019) American Chemical Society.

The importance of non-covalent interactions for the formation of catalytic complexes in lactide polymerization was demonstrated by Romain et al. in a recent publication [131] involved the DFT modeling at the ωB97xD [88]/6-31G(d,p) level of theory of (dl)-LA ROP catalyzed by aluminium complex **51** (Figure 19). The calculations established that the NH hydrogen bond donors are well positioned in the catalytic pocket to interact with the reactive species (lactide, growing polymer) and to form hydrogen bonds (Figure 26). The results of the modeling were confirmed by the fact of low activity of the N-methyl substituted complex and by the kinetic isotope effect arising after the replacement of NH by ND fragment. The modeling of the propagation stage allowed to explain the preference of the formation of heterotactic PLA.

**Figure 27:**
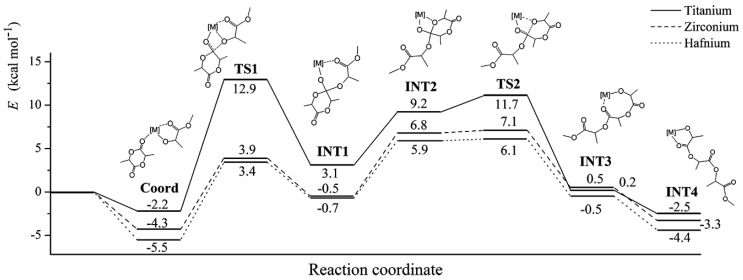
Energy profiles of *l*LA ROP catalyzed by complexes **52**. Reprinted with permission from [132]. Copyright (2014) American Chemical Society.

**Figure 28:**
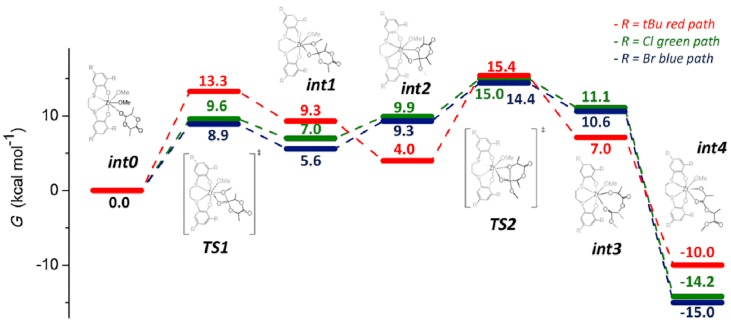
Computed reaction paths for the ring-opening of l-lactide promoted by **53a–c**. Reprinted with permission from [133]. Copyright (2019) Elsevier B.V.

Group IV metal bis(aryloxy) complexes with MeS substituents in the *ortho* position **52** were studied using DFT modeling at the BP86/LANL2DZ level [44,45,143] as a catalysts of l-LA ROP by Milione et al. [132]. The reaction profile obtained (Figure 27) deserves special attention. After the rate-limiting insertion step, the loss of lactate coordination preceeded the ring-opening. A similar reaction pathway was found for LA ROP catalyzed by main-group metal complexes (see below). The closely related bis-phenoxy complexes of zirconium **53** (Figure 19) were studied as l-LA ROP catalysts by Millone et al. [133]. DFT calculations at the BP86/LanL2DZ+6-31G(d) [44,54,78] level were performed for the evaluation of the partial atomic charge of the Zr atom of the optimized structures of **53a**–**c**. Natural population analysis (NPA) revealed that the substitution of *tert*-butyl with chlorine or bromide atoms in the phenolate rings determines an increment of the NBO charge of the Zr atom from +1.48 au in **53a** to +1.52 au in both **53b** and **53c**. The Lewis acidity of the zirconium center has a direct consequence on the monomer binding: the formation of the complexes with l-LA was detected in the case of **53b** and **53c**, the complex **53a** did not form a stable adduct. Also, the increase of the positive charge at the metal center lowers the barrier for the nucleophilic attack of the alkoxide group to the carbonyl group of the monomer (Figure 28). Both studies [132,133] have not considered the propagation stage of l-LA ROP which should be qualitatively different from the initiation stage because of the involvement of chelate complexes INT4 (Figure 13 and Figure 14). 

**Figure 29:**
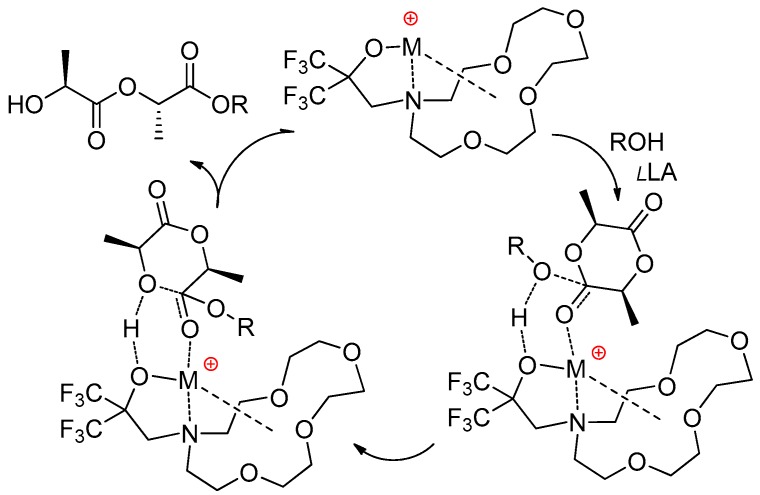
Proposed ligand-assisted activated monomer mechanism for *l*LA ROP mediated by **54**.

Discrete cationic complexes of divalent rare-earth and alkaline-earth metals **54** (Figure 19) were studied in LA ROP [134]. The authors proposed an extended “ligand-assisted activated monomer mechanism” (Figure 29) with the participation of an extended initiator (ROH or hydroxy end-group of the growing PLA chain) and confirmed this assumption by DFT calculations at the B3PW91 [51,52] level. The activation barrier of LA ROP was calculated as 19–22 kcal/mol, a value in good agreement with the experimental result for **54b** (18.3 kcal/mol). The rational design of the ligand in **54** deserves particular attention: high stability and high activity of divalent rare-earth metal complexes was supported by fluoroalkyl-substituted alkoxy fragments, in the absence of fluoroalkyl groups such complexes rapidly formed inactive M (III) species.

DFT modeling of LA ROP catalyzed by Li, Mg and Zn alkoxides **55**–**57** (Figure 19) at the B3LYP/6-31G(d,p) level [51,53,54] was carried out by Galabova et al. [135]. Only the initiation stage was analyzed in this work. The value of CN in intermediates and TS was set to 3 for all metals, but this value seems to be low at least for Mg and Zn. On the other hand, all key transition states have been found and discussed, which enabled to address this work as a useful draft for the modeling of LA ROP catalyzed by main-group metal alkoxides.

As was found in silico (and confirmed experimentally), the LA molecule can be a source of nucleophilic alkoxy fragment in LA ROP catalyzed by zirconium of hafnium carbamates **58** (Figure 19) at elevated temperatures [136]. DFT calculations at the B3LYP/6-31G** [51,53,54] level concluded that the formation of enolate complex is endergonic process with ΔH^≠^ ~ 35 kcal/mol, the insertion and ring-opening of the second LA molecule required overcoming of an activation barrier characterized by the energy of additional 8 kcal/mol in H scale. Unfortunately, the chain propagation stage was not simulated, but it is estimated that the activation energies for this stage should be lower. The presence of enolate end-fragment in PLA chain was detected by NMR spectroscopy. 

As mentioned above, redox-switchable catalyst **35** (Figure 16) exhibits different activities in TMC ROP depending on an oxidation state of Fe in ferrocene moiety [110] (see Section 3). A closely related study of l-LA ROP was implemented by Diaconescu et al. [137] for post-metallocene Zr complex **59** (Figure 19). This study included DFT modeling at the B3LYP/6-31G(d)-LANL2DZ [51,53,54,78] level that resulted in the paradoxical result of the formation of Zr/Fe (III) ring-opened chelate complex from initial chelate formed after first LA insertion with a total change in free energy of +24.7 kcal/mol. This indicates, in our view, a technical error with high probability (for example, incorrect spin state specification). On the other hand, the calculated free activation energy for propagation stage (20.5 kcal/mol) was consistent with experimental observations.

**Figure 30:**
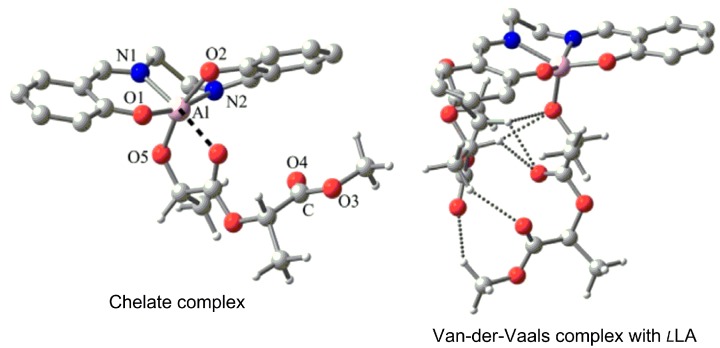
Chelate and more stable Van-der-Vaals complexes based on **18a**. Reprinted with permission from [64]. Copyright (2017) Elsevier B.V.

Significant results were obtained by Chandanabodhi and Nanok [64] for l-LA ROP catalyzed by Al complexes **18** and **19** (Figure 6). Both initiation and propagation stages were modeled thoroughly, the significant difference between alkoxy-Al and lactate-Al initiators ate these stages had been demonstrated. In contrast with other theoretical works that ignored the formation of five-membered chelates, or treated such chelates as a ground states of polymerization due to high stability of similar compounds, the authors demonstrated that such chelates are less stable than the Van-der-Vaals complexes with *l*LA molecule containing η^1^-bonded lactate fragments (Figure 30). 

This finding is of particular interest in the light of the synthesis of random copolymers of lactides with other cyclic substrates.

Lin et al. [138] carried out the modeling of LA ROP catalyzed by zinc alkyl complex **60** (Figure 19) at the B3LYP/6-31G(d)-LANL2DZ [51,53,54,78] level. The mechanism proposed in this study was qualitatively different from traditional coordination-insertion ROP mechanism: the catalyst served as a specific site for the coordination of LA and ROH initiator molecules, the Zn–Et bond was not changed during the reaction. The clear effect of the pendant aryl substituents was also demonstrated, the best catalytic activities were reached for 2-thienyl-substituted complex. 

**Figure 31:**
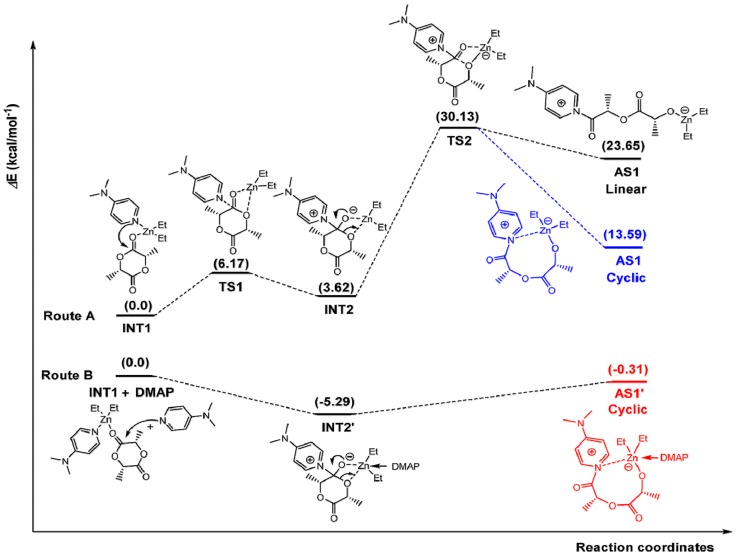
The proposed key species and corresponding calculated energies from different reaction routes of LA ROP catalyzed by **61**. The optimized structure of TS1′ and TS2′ were not obtained. Reprinted with permission from [139]. Copyright (2018) Wiley-VCH Verlag GmbH & Co.

Finally, very interesting results were obtained at the intersection of coordination catalysis and organocatalysis when LA ROP was initiated by ZnEt_2_ and *N*,*N*-dimethyleminopyridine (DMAP) complex **61** [139]. DFT calculations of the stationary points and transition states were performed at the B3LYP [53] level with the 6-31G [144,145,146] and LANL2DZ [78] basis sets for three possible reaction pathways. The preference of partial dissociation of one DMAP molecule was demonstrated, and the complex containing coordinated LA and DMAP molecules was subjected to nucleophilic attack of the free DMAP molecule with a formation of ring-opened zwitterionic acyl complex; it is the participance of the second DMAP molecule that lowering the activation energy of ROP (Figure 31).

### 4.2. Binuclear ROP Mechanism

**Figure 32:**
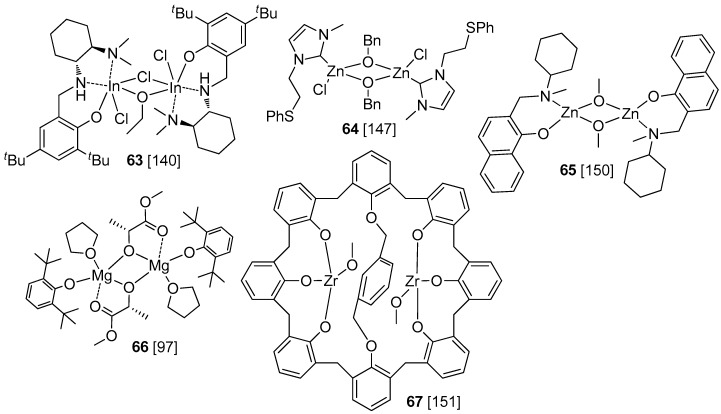
Binuclear metal complexes studied in DFT modeling of lactide ROP [97,140,147,150,151].

As demonstrated in a number of publications, heteroleptic metal alkoxides form stable binuclear complexes [69,94,97,99,140,147,148,149] that are able to catalyze ROP of cyclic esters by both binuclear and mononuclear mechanisms. DFT modeling has been shown to be an effective instrument of choice of the preferable reaction pathway. The first comparative theoretical study of mononuclear and binuclear LA ROP mechanisms was performed by Maron et al. for indium complexes **62** (Figure 19) and **63** (Figure 32) [140] at the B3PW91 [51,52] level of theory. The preference of binuclear pathway was proposed on account of high dissociation energy of **63**, and the results of thorough theoretical analysis of the stereochemistry of (dl)-LA ROP were in good agreement with the experimental data. We also note that In catalyst **63** did not demonstrated the propensity to form chelate complexes that plays significant role in Mg-catalyzed binuclear catalytic process [97] (see below).

**Figure 33:**
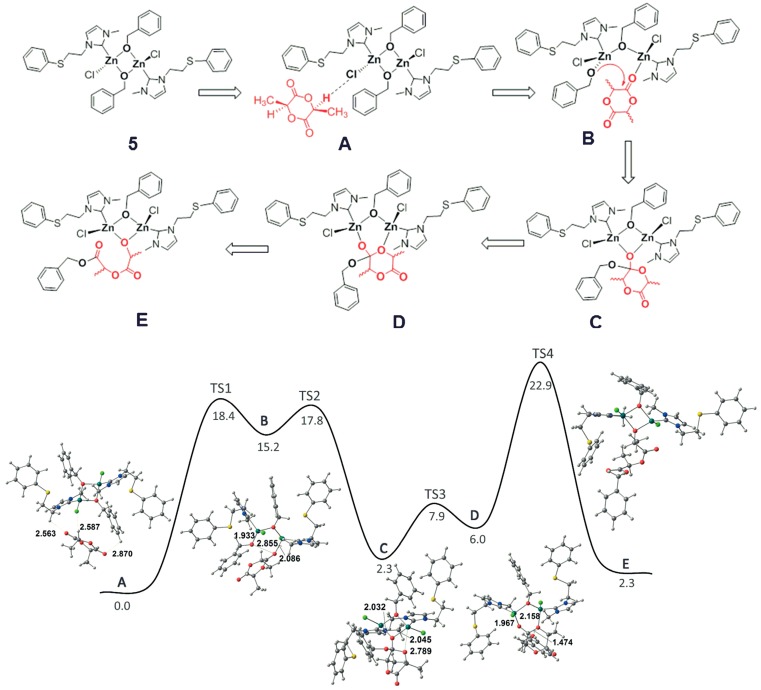
Relevant intermediates in *l*LA ROP catalyzed by complex **64** (top); energy profile for the first stage in *l*LA ROP catalyzed by complex **64** (Gibbs free energies in kcal/mol) and structures of the intermediates (bottom). Reprinted with permission from [147]. Copyright (2014) Wiley-VCH Verlag GmbH & Co.

A similar pattern (reaction mechanism without the formation of highly stable chelates) was detected by Dagorne et al. who studied l-LA ROP, catalyzed by dimeric NHC zinc complex **64** (Figure 32) [147]. DFT calculations were performed at the PBE0 [142] level of theory using Stuttgart effective core potentials and their associated basis set [107] for zinc and the 6-31G** basis [144,145,146] for other atoms. Similarity of the reaction mechanisms of the initiation stage (Figure 33) and propagation stage was attributable to the lack of lactate chelation after ring-opening of the first LA molecule. The free activation energy was calculated as a relative free energy of the rate-limiting ring-opening step TS and was 22.9 kcal/mol. Thus, the catalyst can be regarded as highly active for l-LA ROP. What is most interesting is the ability of **64** to catalyze PLA degradation by MeOH under mild conditions to methyl lactate and higher oligomers. 

**Figure 34:**
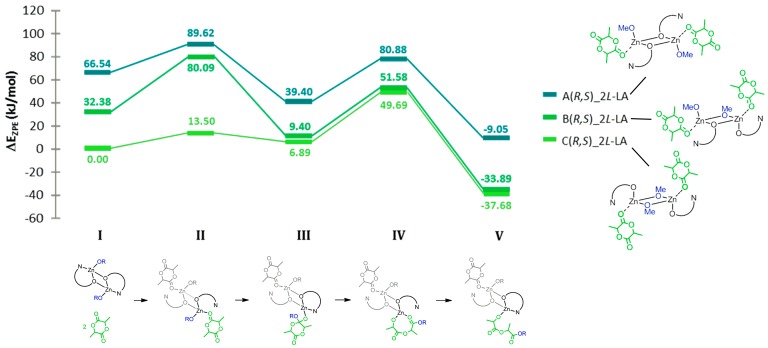
Energy profile for the first cycle in ROP of lactide initiated by zinc compounds based on **65**. Reprinted with permission from [150]. Copyright (2017) American Chemical Society.

The structure and catalytic properties of Zn complexes **65** with chelating NO ligands were explored thoroughly by Ejfler et al. [150]. This complex and related compounds with *tert*-butylphenyl fragments in place of naphthyloxy group demonstrated dynamic solution behaviour with a formation of mononuclear ZnL_2_ species and tri- and tetra-Zn complexes. This equilibrium was studied by DOSY NMR, the relative stability of mononuclear and oligonuclear species was estimated using DFT modeling at the B3LYP/6-31G* [51,53,54] level of theory. Much more interesting dynamic processes in the ligand environment of Zn atoms were explored using DFT calculations of the reaction profiles of LA ROP. At the initiation stage, the reaction pathway involving the complex A with two isolated MeO fragments (Figure 34) turned out to be the most favorable. Note that ring-opened LA fragment did not form stable five-membered chelate complex with Zn atom similarly to **64** [147].

**Figure 35:**
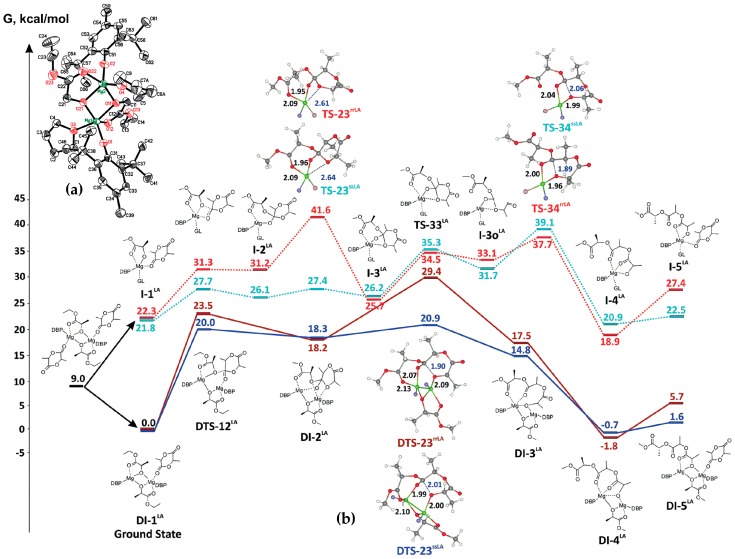
(**a**) Molecular structure of the prototype of the complex **66** (X-ray diffraction data); (**b**) Reaction profiles of lactide ROP for mononuclear and binuclear coordination–insertion mechanisms. Geometries of higher energy TS are shown; DBP and GL fragments are omitted for clarity, phenolate and GL oxygen atoms are colored in blue and pink, correspondingly. Reprinted with permission from [97]. Copyright (2017) Royal Society of Chemistry.

Such chelate formation plays a key role in (dl)-LA ROP catalyzed by binuclear BHT-Mg complex **66** [97]. The structure of the complex **66** used for DFT modeling was obtained by minor simplification of the molecular structure of ethyl lactate complex which had been determined by X-ray diffraction analysis (Figure 35a). We analyzed the reaction profiles for binuclear complex **66** and corresponding mononuclear species containing glycolide (GL) molecule to fill the coordination sphere of Mg atom. The comparative DFT calculations were performed at the B3PW91/DGTZVP [52,98] level of theory, taking into account the endergonic character of the dissociation of **66**. The results of the modeling confirmed the preference of the binuclear pathway and allowed us to explain the experimental fact of heterotactic PLA formation in (*dl*)LA ROP, catalyzed by dimeric BHT-Mg complexes.

Another significant factor arising from the binuclear nature of the catalysts was the formation of the macrocyclic polymerization products. The related processes were detected and explained using DFT modeling when **67**-catalyzed (dl)-LA ROP was studied [151]. **67** was a stable binuclear complex with 1,5-bridged-calix[8]arene ligand (Figure 32) that demonstrated moderate catalytic activity at 100 °C (TOF ~200 h^−1^). DFT calculations were carried out to prove that the two metal centers in **67** favor the chelation of the growing polymer chain and facilitate the formation of lactyl macrocyclics. The folding of the polymer chain in binuclear complex was predicted to be exergonic by −6.9 kcal/mol, while such process in model calix[4]arene mononuclear complex was found to be slightly endergonic (+3.7 kcal/mol). The results of the modeling were confirmed by preferable formation of macrocyclic PLA in polymerization catalyzed by calix[8]arene-based dizirconium complex.

In conclusion, we should like to emphasize that some aspects of the molecular structure of ROP precatalysts have been refined by DFT modeling [148,152,153,154,155,156,157]. However, the results of such modeling do not reflect fully the catalytic behavior of these complexes and therefore were irrelevant for our review.

## 5. Coordination Polymerization of Related Compounds. Ethylene Phosphates

It is no wonder that the most of publications on the topic of DFT modeling of ROP of cyclic esters focus on the processes involving conventional cyclic esters such as εCL, l-LA, (dl)-LA, TMC due to high practical importance of the corresponding polymers. In addition to these articles, discussed above, only two papers have addressed questions of ROP of structural analogs of εCL and δVL, namely, 1,4-dioxepan-5-one [119] and 1,4-dioxan-2-one (*para*-dioxanone, PDO) [70]. DFT modeling of 1,4-dioxepan-5-one ROP, catalyzed by Sn(OAc)_2_ (**36**, Figure 6) with MeOH initiator, was performed in the framework of the reaction mechanism, presented in Figure 20, and resulted in estimation of the activation energy by the value of 23.1 kcal/mol; nucleophilic attack of the alkoxy initiator was found to be a rate-limiting step. Low-level comparative modeling of ROP of lactones, TMC and PDO, catalyzed by [BHT-Mg-OMe] catalyst **25a** (Figure 6), allowed us to rank cyclic esters by reactivity as: PDO > δ-VL > TMC ~ ε-CL [70].

In recent years the list of prospective ROP substrates was significantly enhanced by cyclic esters of phosphoric and phosphinic acids [12,13,158,159,160]. Five-membered cyclic alkyl ethylene phosphates (ROEP) and alkyl ethylene phosphonates (REP) demonstrated excellent reactivity in ROP, both coordination catalysts and organocatalysts have been successfully applied in the synthesis of the corresponding polymers. However, the theoretical studies of ROEP polymerization have been started just last year [161,162].

**Figure 36:**
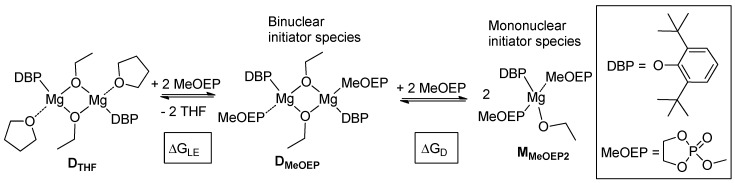
Ligand exchange and dissociation for **24b** with the formation of dimeric and monomeric initiator species [161].

In 2018, we performed DFT modeling of ROP of methyl ethylene phosphate (MeOEP, Scheme 1, R = Me in ROEP formula), catalyzed by binuclear magnesium complex **24b** (Figure 6) [161,163]. The calculations were carried out at the B3PW91/DGTZVP [52,98] level of theory for the comparison of the results obtained for MeOEP with the results of the modeling of εCL and (dl)-LA ROP [97]. The key issue was the nature of the catalytic species that may represent both binuclear and mononuclear complexes. We calculated the free energy of dissociation for binuclear complex formed by **24b** backbone and MeOEP molecules instead of THF (Figure 36) and found that the formation of a monomeric complex was energetically unfavorable, ΔG_D_ = 10.1 kcal/mol.

**Figure 37:**
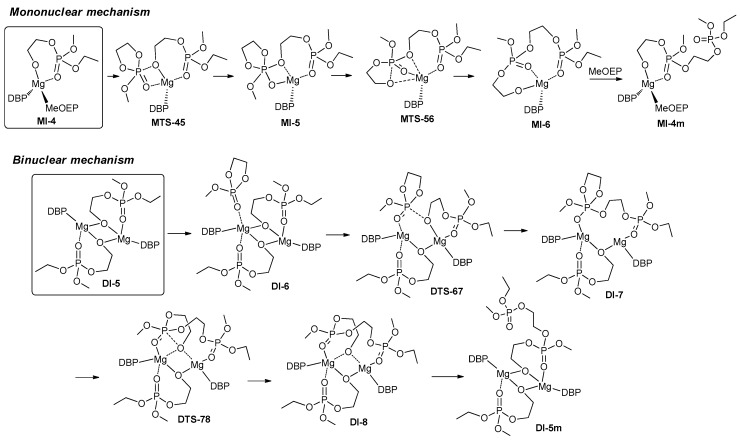
Intermediates and transition states of the propagation stage for mononuclear (**top**) and binuclear (**bottom**) mechanisms of MeOEP ROP [161].

However, this value was not sufficient to unambiguously determine the preferred reaction mechanism, and we made detailed calculations for both possible reaction pathways. The modeling of the initiation stage allowed us to determine the structures of the ground states MI-4 and DI-5 that were used as a starting stationary points in the modeling of the propagation stage for mononuclear and binuclear mechanisms, respectively (Figure 37).

**Figure 38:**
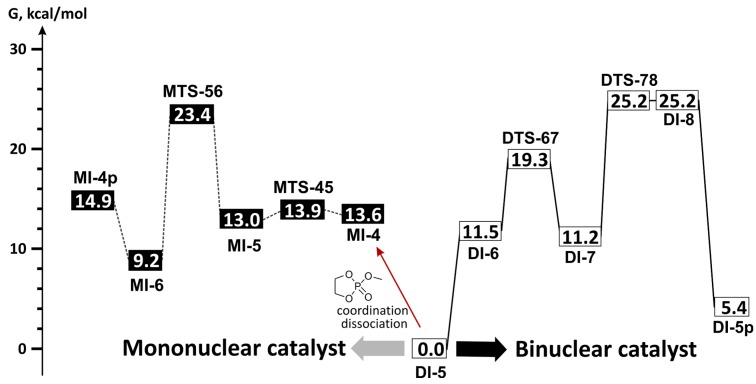
Energy profiles of BHT-Mg catalyzed ROP of MeOEP for the mononuclear (---- dark gray, left) and binuclear (**—** black, right) reaction mechanisms [161].

The free activation energy of MeOEP ROP catalyzed by mononuclear complexes was approximately 14 kcal/mol, and the activation barrier for the binuclear mechanism was 25.2 kcal/mol. To establish the preferred reaction pathway, we calculated the free energy change in the formation of MI-4 by additional coordination of MeOEP and dissociation of the binuclear complex DI-5. Taking into account this difference (13.6 kcal/mol_Mg_), we compared the reaction profiles of the propagation stages for the mononuclear and binuclear mechanisms (Figure 38). This comparison allowed us to draw a conclusion about the preference of mononuclear reaction pathway for MeOEP ROP, catalyzed by BHT-Mg complexes. This finding was supported by the results of the experiments in the synthesis of poly(MeOEP)-*b*-poly(l-LA) and poly(MeOEP)-*b*-poly((dl)-LA) that demonstrated the formation of highly active mononuclear catalytic species through prepolymerization of MeOEP. The results of DFT modeling of MeOEP ROP were then used in DFT modeling of MeOEP/LA random copolymerization (see below).

## 6. Coordination Random Copolymerization

Clearly, the DFT modeling of random copolymerization is several times more difficult than the modeling of homopolymerization. Such modeling must address all possible comonomer sequences bearing in mind the chemical difference of both cyclic substrates and corresponding ring-opening products.

**Figure 39:**
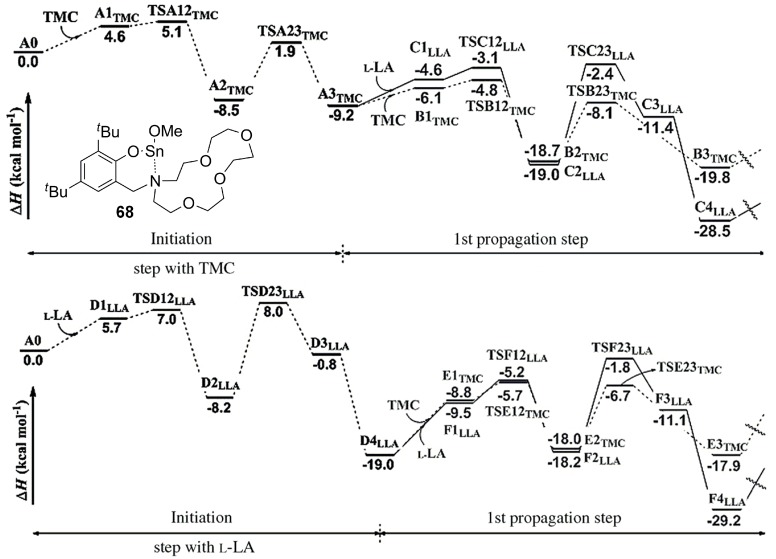
Calculated reaction pathways for the initiation and first propagation stages of ROP of TMC (**top**) and *l*LA (**bottom**) mediated by the complex **68**. Reprinted with permission from [164]. Copyright (2013) Wiley-VCH Verlag GmbH & Co.

A top-quality investigation was performed by Sarazin et al. for the series of Sn (II) heteroleptic complexes with modified bulky aryloxy ligands [164]. DFT calculations at the B3PW91 [51,52] level, using the 6-31G(d,p) [79] and the SDD [107] basis sets, were performed for TMC and l-LA ROP catalyzed by model complex **68** (Figure 39). DFT calculations shown that, out of the four possible monomer insertion sequences, (1) TMC then TMC, (2) TMC then l-LA, (3) l-LA then l-LA, and (4) l-LA then TMC, the first three were possible. By contrast, insertion of l-LA followed by that of TMC was endothermic by +1.1 kcal/mol. The analysis of the reaction profiles (Figure 39) allowed the authors to draw a conclusion about thermodynamic control in TMC/l-LA copolymerization; the key structural factor was the loss of κ^2^-lactate coordination in the case of “l-LA then TMC” comonomer sequence with a formation of less stable κ^1^-alkoxy complex. The results of calculations were in good agreement with copolymerization experiments: introduction of 1:1 TMC/*l*LA mixtures resulted in the formation of copolymers with minimal TMC rate.

**Figure 40:**
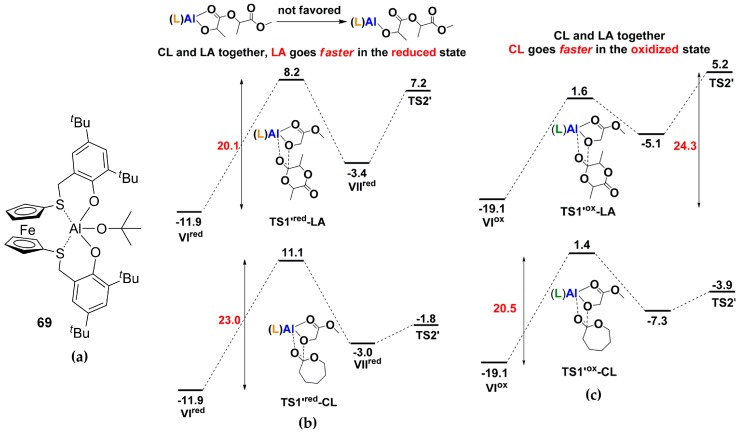
(**a**) The molecular structure of **69** (reduced form); Potential energy surfaces of the propagation steps with **69** in Fe (II) (**b**) and Fe (III) (**c**) forms. Reprinted with permission from [165]. Copyright (2017) American Chemical Society.

The significance of κ^2^-lactate coordination in εCL/l-LA copolymerization had been demonstrated by Diaconescu et al. who studied aluminium complexes **69** with redox-switchable ferrocene-based OSSO ligand [165]. DFT calculations, performed at the B3LYP/6-31G(d)-LANL2DZ [51,53,54,78] level of theory, demonstrated a determining influence of the oxidation state of Fe in ferrocene moiety on energy reaction profiles for l-LA/l-LA and l-LA/εCL comonomer sequences (Figure 40). However, the formation of κ^2^-lactate complexes continued to serve as a key factor of the ROP thermodynamics, the formation of random lactide/lactone copolymers was failed.

In the study of terpolymerization of lactide, epichlorohydrin (ECH) and phthalic anhydride (PA), catalyzed by aluminium complex **49** (Figure 19), activated by [Ph_3_P=N=PPh_3_]Cl, Pand, Chen et al. [166] performed a theoretical analysis of the formation of active metal species, initiation of ROP, and chain propagation for all comonomer sequences at the M06-D3/6-311+G(d,p)//B3LYP/6-31G(d) level of theory [51,53,54,85]. DFT calculations indicated that the copolymerization of ECH and PA was more kinetically favorable than LA homopolymerization at the earlier stage, but after consumption of PA, the homopolymerization of LA rather than LA/ECH copolymerization could occur.

**Figure 41:**
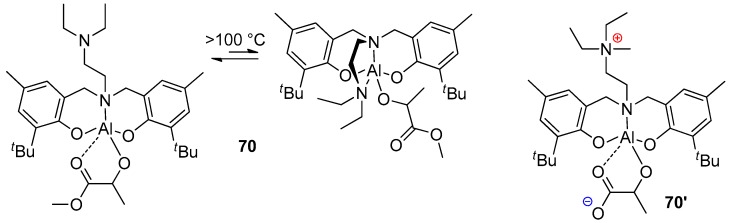
κ^2^-Lactate–κ^1^-alkoxy transformation of the complex **70** at elevated temperatures, and inactive complex **70’** formed by side reaction [167].

Apparently, specific ligand structure with donor side-arm allows to come close to the target of the synthesis of random lactone/lactide copolymers. Phomphrai et al. [167] carried out a study of the catalytic behaviour of the complex **63** and came to the conclusion that κ^2^-lactate fragment is undergoing transformation to κ^1^-alkoxy fragment at elevated temperatures (Figure 41). Specifically to complex **70**, such process can be complicated by the side reaction of alkyl migration to nitrogen atom. DFT modeling of this reaction has led to the view that the mechanism of this reaction is bimolecular, and the product is zwitterionic catalytically inactive complex **63’** (Figure 41).

**Figure 42:**
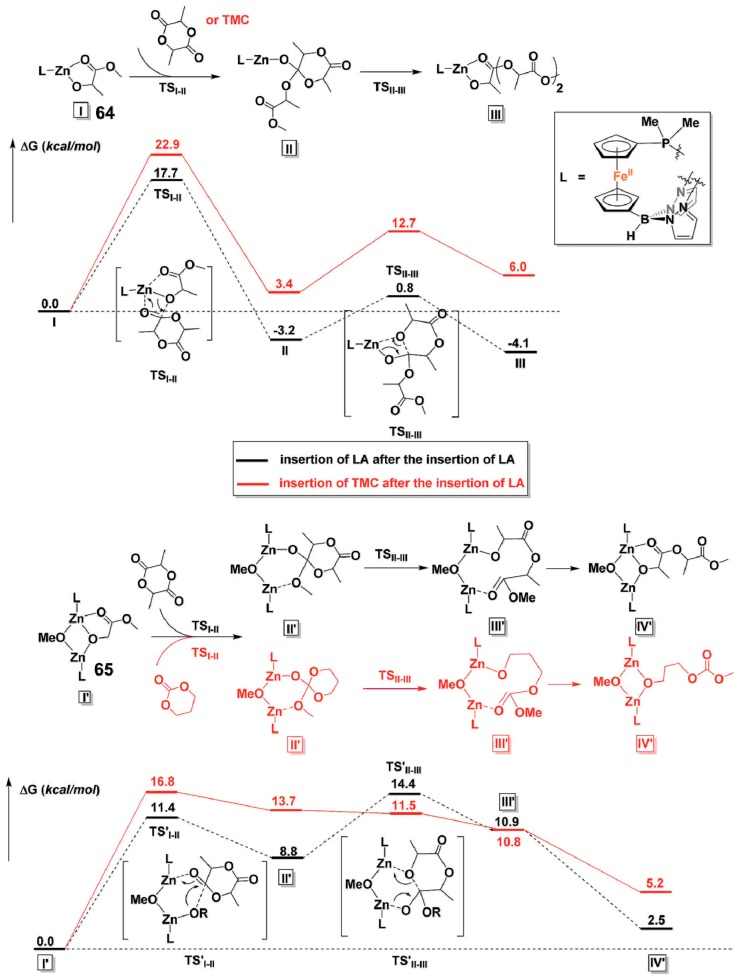
Comparison of reaction coordinates for propagation catalyzed by a monomeric (**71**) or dimeric (**72**) zinc complexes. Reprinted with permission from [168]. Copyright (2018) Royal Society of Chemistry.

Copolymerization of TMC and *l*LA, catalyzed by Zn complexes **71** and **72** (Figure 42) was studied by Diaconescu [168] experimentally and theoretically at the B3LYP/6-31G(d)-LANL2DZ [51,53,54,78] level.

Strictly speaking, this study considered the synthesis of multiblock, not random, TMC/*l*LA copolymers, but at the same time it involved thorough comparative analysis of the chain propagation stage by l-LA or TMC in mononuclear **71** and binuclear **72** κ^2^-lactate complexes. The comparative analysis of the reaction profiles (Figure 23) indicated that: (1) in solution, the dimeric **65** species was more favored than the monomeric **71** species; (2) the dimeric **72** species had lower overall activation barriers compared to the monomer; (3) both the polymerization of l-LA and TMC were possible with the dimeric **72** and the rate of the polymerization of TMC is faster than that of l-LA; (4) however, after the insertion of l-LA, the insertion of another l-LA was easier than the insertion of TMC.

Very recently [141], we performed DFT modeling of copolymerization of εCL, l-LA and MeOEP in twinning combinations, catalyzed by binuclear and mononuclear DBP-Mg complexes **25b** (Figure 6), **50** (Figure 19) and **66** (Figure 32). The calculations were performed at the B3PW91/DGTZVP level of theory for comparison with previously reported results [97,161]. We used binuclear mechanistic concept for simulations of εCL/l-LA ROP, and mononuclear mechanistic concept in modeling of εCL/MeOEP and l-LA/MeOEP copolymerization. The results of the modeling allowed us to conclude on possibility of random l-LA/MeOEP copolymerization, this assessment was confirmed by the preparation of copolymers. In addition, we found experimentally and confirmed theoretically, that poly(MeOEP) chains may be subject to transesterification under the mild reaction conditions. This process resulted in increase of copolymer statisticity.

In summary, the results of DFT modeling of random copolymerization of cyclic substrates of different chemical nature allowed to identify the principal challenge in the synthesis of highly statistical copolymers with given comonomer ratios. This challenge arises from the difference in relative stability of the complexes formed by ring-opened lactones or carbonates (κ^1^-alkoxy complexes) and ring-opened lactides or phosphates (more stable κ^2^-lactate or κ^2^-alkoxyphosphate complexes). The results of experimental investigations and theoretical analysis of copolymerization mechanisms are already providing possible ways for the development of such catalysts. The first prospective way may be based on the control of the electrophilicity of the metal center with the purpose to mitigate the donor properties of different cyclic substrates and to decrease the difference in relative energies of κ^1^-alkoxy and κ^2^-lactate or κ^2^-alkoxyphosphate complexes. The second possible way of the catalyst design would be to use the specific polydentate ligands containing donor atom in the side group, that is capable for reversible coordination at metal atom that results to partial loss (κ^2^- to κ^1^-) of polymeryl chain-end coordination, as was demonstrated by Phomphrai et al. in [167]. In doing so, the possibility of the reaction between ligand and coordinated polymeryl end-group should be considered.

## 7. Concluding Remarks

**Figure 43:**
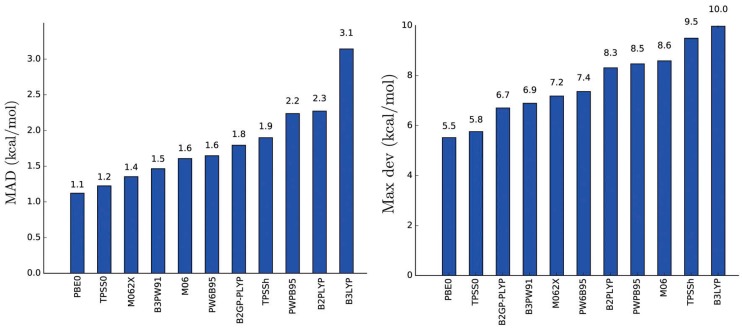
The mean average deviation (**left**) and maximal deviation (**right**) of the 24 computed stationary states, with respect to the E_comp_ energy reference. Reprinted with permission from [169]. Copyright (2017) Royal Society of Chemistry.

From a technical standpoint, the reliability of the DFT calculations is directly dependent on the level of the theory used. Unfortunately, only two publications contained comparative analysis of different functional/basis combinations used in DFT modeling of ROP. The first study [161] compared deviations of MeOEP geometry from experimental data taking into account the calculation time. The second [169] discussed the results of high-level ab initio modeling of ROP of cyclic esters, catalyzed by [Me_2_AlOMe], in comparison with the results of DFT calculations using different functionals (Figure 43). It was noted that PBE0 had the best performance in terms of relative energies, when comparing it to the ab initio results; the commonly used B3LYP functional demonstrated the worst characteristics.

The neglect of the reaction conditions (solvent, temperature etc.) in certain studies was a second source of misperceptions. However, in our opinion, the formation of proper mechanistic model is of greater importance than technical details of calculations. The primary mistake in DFT simulations of ROP was an estimation of the polymerization rate using thermochemical data calculated for the initiation stage. Moreover, the lack of understanding the difference between initiation and propagation stages often resulted in missing of the true geometries of stationary points and transition states of ROP. Such inaccuracies devalued the usefulness of DFT modeling as a powerful tool of catalyst and monomer design.

We should be aware that DFT modeling of coordination ROP of cyclic esters represents a convenient method for the explanation, interpretation and visualization of the *experimental* results. Every high-quality DFT simulation of ROP is really an object of art, the makeover of the solid experimental work, but “modeling for modeling” is not making a lot of sense. 

**Figure 44:**
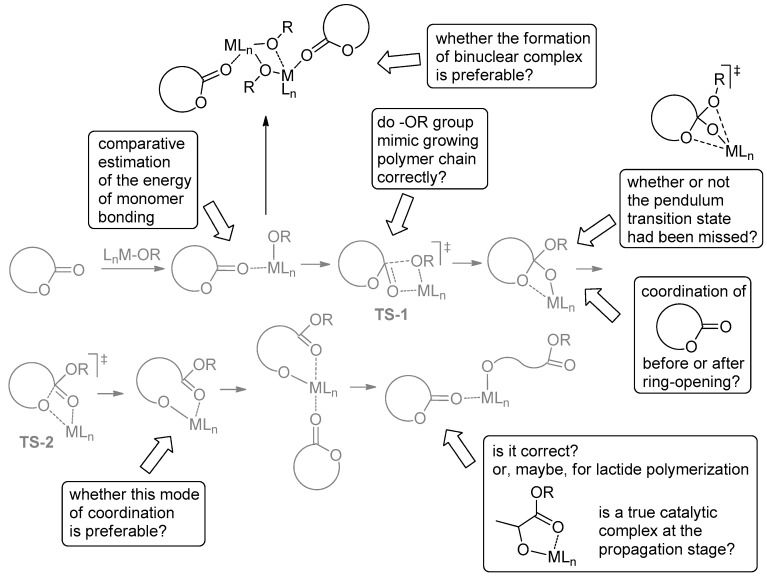
The extension of coordination-insertion ROP mechanisms.

Unfortunately, there is no comprehensive and conventional model of the ROP mechanism, and there cannot be because of a wonderful variety of catalysts and ROP substrates. The fundamental problem of the development of powerful structure-activity relationship for effective design of the catalyst for given cyclic substrate is still far from being resolved. Nevertheless, some fundamental results have been obtained by researchers, in particular, the finding of the correct reaction pathways based on ligand environment and ring-opening product coordination without the missing of valuable transition states (such as pendulum TS discussed in the review), the possibility of the binuclear ROP mechanisms with an explicit cooperative effect, etc. In Figure 44, which represents an update of Scheme 2, we pose some significant questions to be answered prior to and during DFT modeling of ROP.

A rapid progress in computational methods and techniques allows for consideration of the DFT modeling as a common and useful tool for any contemporary investigation of novel ROP coordination catalysts, monomers, processes and product architectures, but such a modeling requires a truly scientific approach bearing in mind the diversity of the species that may be formed in catalytic processes, and the variety of the possible interactions and reactions.
